# Cholesterol reduction by immunization with a PCSK9 mimic

**DOI:** 10.1016/j.celrep.2024.114285

**Published:** 2024-05-30

**Authors:** Baoshan Zhang, Gwo-Yu Chuang, Andrea Biju, Daniel Biner, Jiaxuan Cheng, Yiran Wang, Saran Bao, Cara W. Chao, Haotian Lei, Tracy Liu, Alexandra F. Nazzari, Yongping Yang, Tongqing Zhou, Steven J. Chen, Xuejun Chen, Wing-Pui Kong, Li Ou, Danealle K. Parchment, Edward K. Sarfo, HaoMin SiMa, John-Paul Todd, Shuishu Wang, Ruth A. Woodward, Cheng Cheng, Reda Rawi, John R. Mascola, Peter D. Kwong

**Affiliations:** 1 Vaccine Research Center, NIAID, National Institutes of Health, Bethesda, MD 20892, USA; 2 Research Technologies Branch, NIAID, National Institutes of Health, Bethesda, MD 20892, USA; 3 Aaron Diamond AIDS Research Center, Columbia University Vagelos College of Physicians and Surgeons, New York, NY 10032, USA; 4 These authors contributed equally; 5 Lead contact

## Abstract

Proprotein convertase subtilisin/kexin type 9 (PCSK9) is a plasma protein that controls cholesterol homeostasis. Here, we design a human PCSK9 mimic, named HIT01, with no consecutive 9-residue stretch in common with any human protein as a potential heart attack vaccine. Murine immunizations with HIT01 reduce low-density lipoprotein (LDL) and cholesterol levels by 40% and 30%, respectively. Immunization of cynomolgus macaques with HIT01-K21Q-R218E, a cleavage-resistant variant, elicits high-titer PCSK9-directed antibody responses and significantly reduces serum levels of cholesterol 2 weeks after each immunization. However, HIT01-K21Q-R218E immunizations also increase serum PCSK9 levels by up to 5-fold, likely due to PCSK9-binding antibodies altering the half-life of PCSK9. While vaccination with a PCSK9 mimic can induce antibodies that block interactions of PCSK9 with the LDL receptor, PCSK9-binding antibodies appear to alter homeostatic levels of PCSK9, thereby confounding its vaccine impact. Our results nevertheless suggest a mechanism for increasing the half-life of soluble regulatory factors by vaccination.

## In brief

Proprotein convertase subtilisin/kexin type 9 (PCSK9) controls cholesterol homeostasis. To assess the feasibility of a PCSK9-based heart attack vaccine, Zhang et al. create a PCSK9 mimic and demonstrate in mice and NHPs its ability to significantly reduce cholesterol. Unexpectedly, the vaccine-induced antibodies also altered the PCSK9 half-life, blunting vaccine impact.

## Graphical Abstract

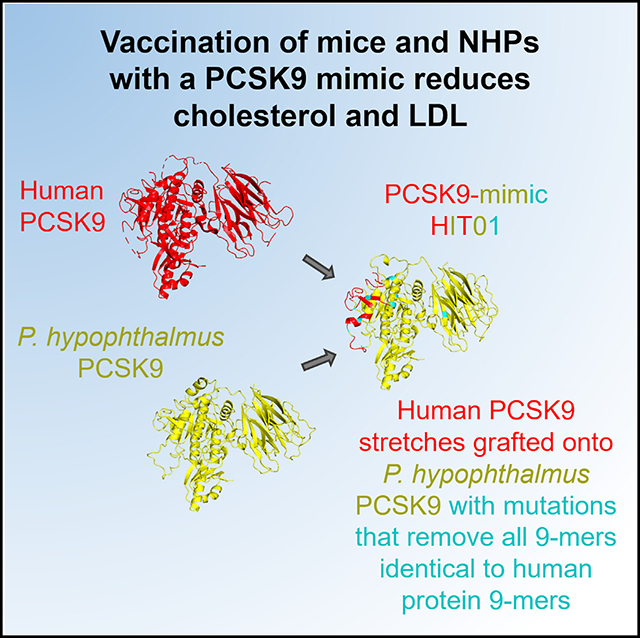

## INTRODUCTION

As a central regulator of plasma low-density lipoprotein cholesterol (LDL-C) levels, proprotein convertase subtilisin/kexin type 9 (PCSK9) has been a successful target of monoclonal antibody therapy to treat hyperlipidemia, with evolocumab (also called AMG145) and alirocumab approved by the United States Food and Drug Administration for human use in 2015.^1–4^ PCSK9 is a soluble protein secreted by the liver that regulates LDL-C levels by down-regulating the LDL receptor (LDLR) on the surface of hepatic cells.^5,6^ PCSK9 binds to LDLR on the cell surface, and the PCSK9-LDLR complex is endocytosed and directed to the endosome/lysosome, leading to the degradation of LDLR. In the absence of PCSK9, LDL-LDLR complexes are directed to the endosome/lysosome, where LDL is degraded, but LDLR recycles to the cell surface, where it binds more LDL and the cycle is repeated, resulting in lower LDL levels. Thus, by inducing LDLR degradation, PCSK9 increases cholesterol levels and is a target for the treatment of hyperlipidemia (Figure S1).

Several interventions are currently available for treating hyperlipidemia by reducing expression of PCSK9^7–11^ or blocking its interaction with LDLR. Monoclonal antibodies (e.g., evolocumab and alirocumab) inhibit PCSK9 interaction with LDLR. Treatment with PCSK9-binding antibodies, in combination with statins, reduces LDL-C levels by as much as 40%–60%.^9,12,13^ However, passive delivery of antibody can require monthly injections. In addition to monoclonal antibodies, a small interfering RNA drug targeting PCSK9, inclisiran, has also been approved for treating cardiovascular diseases by lowering LDL-C levels in patients.^14,15^ Inclisiran functions by reducing the expression of PCSK9 and is administered subcutaneously at the beginning of treatment, again after 3 months, and thereafter every 6 months. These available treatments, while showing efficacy in lowering LDL-C levels, are expensive.

Could a PCSK9 “heart attack” vaccine offer a long-term and cost-effective solution for controlling LDL-C levels? Here, we designed human PCSK9-mimicking immunogens by transplanting antibody- and LDLR-binding epitopes on PCSK9 to PCSK9 homologs from other species. To avoid T cell activation against self-proteins, we eliminated all consecutive 9-residue sequences overlapping with human proteins. We evaluated the PCSK9 mimic in vaccinations of both mice and non-human primates (NHPs). Our results demonstrate that a PCSK9 mimic can elicit PCSK9-specific antibodies and significantly reduce LDL levels.

## RESULTS

### Design of a human PCSK9 mimic, HIT01, with no consecutive 9-residue stretch found in any human protein

**Figure 1.**
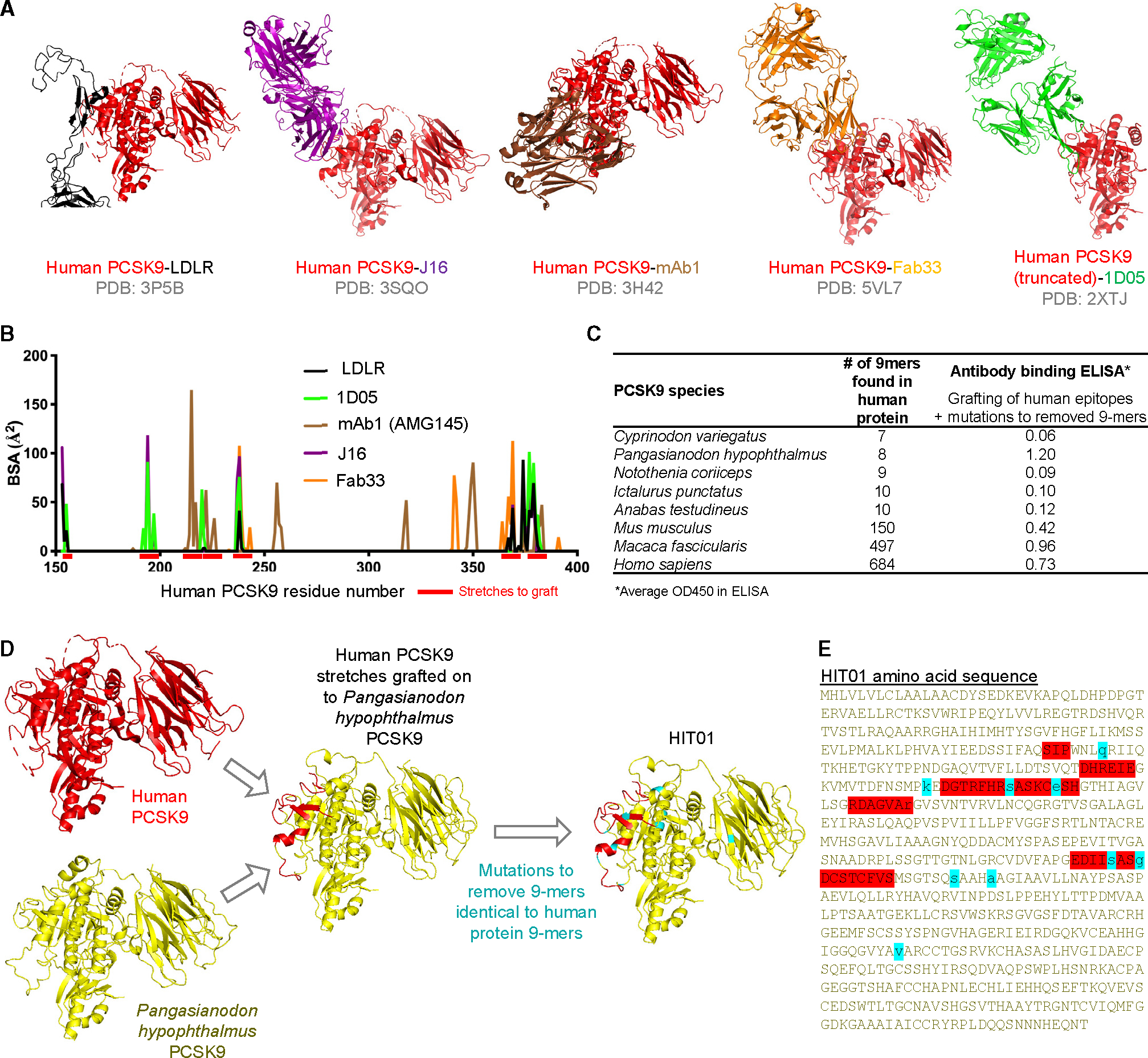
Design of a human PCSK9 mimic, HIT01, with no consecutive 9-residue stretch found in any human protein (A) Published crystal structures of human PCSK9 in complex with LDLR and various therapeutic antibodies, respectively. PBD codes are listed under each structure. (B) Buried surface area of PCSK9 residues to LDLR and various therapeutic antibodies, respectively. (C) Non-human PCSK9 with the least number of 9-mers identical to 9-mers found in human proteins. Antigenicity values are geometric mean ELISA-binding signals for the five antibodies shown in Table S1. (D) Schematic of design PCSK9-mimic HIT01 by grafting the human PCSK9 residue stretches on to *Pangasianodon hypophthalmus* (iridescent shark) PCSK9 and removing 9-mers identical to human protein 9-mers. (E) Sequence of HIT01. Red color indicates residues of antibody epitopes transplanted from human PCSK9. Cyan color indicates an alteration to remove 9-mers identical to human protein 9-mers. See also Figures S1 and S4 and Table S1.

To design a mimic of PCSK9, with no consecutive 9-residue stretch in common with any human protein, we examined the structures of human PCSK9 in complex with the LDLR^16,17^ (PDB: 3P5B) as well as with antibodies reported to reduce cholesterol levels when passively infused (PDB: 3H42, 3SQO, 5VL7, and 2XTJ)^18–21^ (Figure 1A; Figure S1). By examining the overlap in recognized residues of PCSK9, we selected seven residue stretches that were critical for recognition (Figure 1B). We grafted these residues onto divergent PCSK9s, which, prior to grafting, had a handful of 9-mer peptide fragments in common with human proteins. We examined the location of these 9-mer fragments, introduced point mutations based on structural analysis and divergence of PCSK9-species variants, and assessed recognition with antibodies reported to reduce cholesterol (Figure 1C; Table S1). The highest recognition was observed for grafting of the human PCSK9 onto iridescent shark (*Pangasianodon hypophthalmus*, GenBank: KAB5539765.1; Figure S4) PCSK9 with an optical density (OD)_450_ of 2.3, and we further removed 9-mer stretches by mutagenesis to arrive at a version of iridescent shark PCSK9 with no 9-mers in common with any human protein that still bound well to all five antibodies (OD_450_ of 1.2) (Figures 1D and 1E; Table S1). We named the iridescent shark PCSK9 with no 9-mers in common with any human protein “HIT01.”

### Murine immunization with human PCSK9 mimic HIT01 significantly reduces cholesterol and LDL concentration

**Figure 2.**
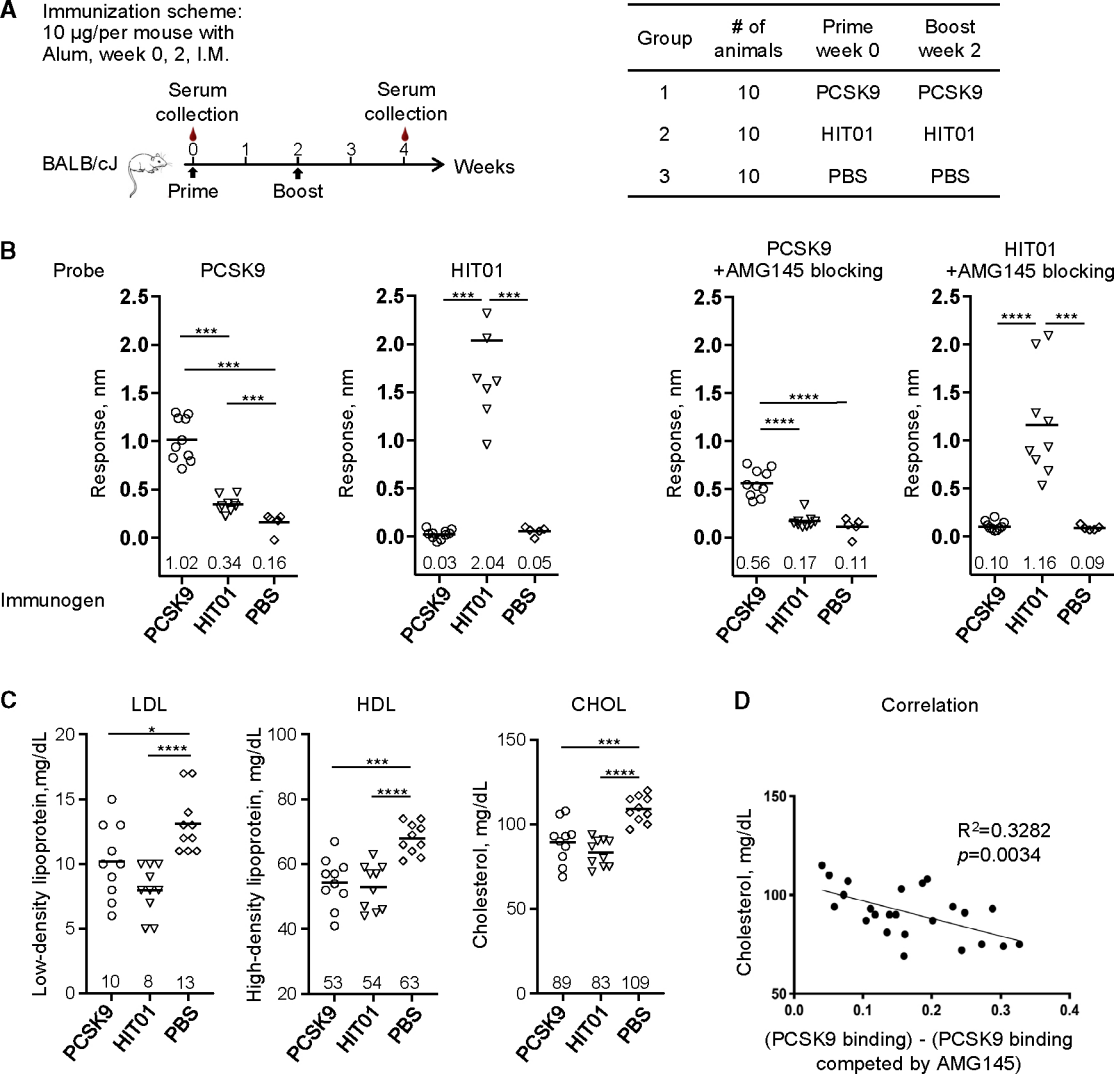
Murine immunization with human PCSK9 mimic HIT01 significantly reduces CHOL and LDL concentration (A) Mouse immunization scheme and animal group information. Each group of 10 mice was immunized two times. Sampling times are indicated on the scheme. (B) Sera BLI binding response against human PCSK9 and HIT01 probes and in the presence of AMG145 blocking on the two probes. Numbers above the x axis are serum antibody response mean values for each group. *p < 0.05, *p < 0.01, ***p < 0.001, and ****p < 0.0001 (Mann-Whitney test). (C) Sera concentration of LDL, total cholesterol (CHOL), and high-density lipoprotein at week 4. Numbers above the x axis are mean values for each group. *p < 0.05, **p < 0.01, ***p < 0.001, and ****p < 0.0001 (Mann-Whitney test). (D) Correlation versus cholesterol of serum responses targeting AMG145 epitope, as defined by PCSK9-binding ELISA minus PCSK9-binding ELISA when competed by antibody AMG145. See also Figure S5.

To evaluate HIT01 as a vaccine immunogen, we immunized BALB/cJ mice (*n* = 10 per group) with human PCSK9 and HIT01 proteins formulated with Alum adjuvant (Figure 2A). Another group of 10 mice were immunized with PBS buffer and adjuvant as a control. All animals were immunized at weeks 0 and 2, and blood samples were collected at 2 weeks after the second immunization. Serum samples were evaluated by Octet using human PCSK9 and HIT01 as probes (Figure 2B). The human PCSK9 immunogen elicited the highest antibody responses against human PCSK9 among the three groups. HIT01 elicited the highest antibody responses among the three groups against HIT01. HIT01 induced significantly higher responses to human PCSK9 than PBS control, but there was no significant difference for responses to HIT01 between PCSK9 and PBS groups. We also measured mouse serum antibody reactivity to mouse PCSK9. As expected, the serum antibody responses against mouse PCSK9 were lower than the responses to the HIT01 immunogen (Figure S5). We next examined whether elicited responses competed with AMG145,^22–25^ a well-characterized monoclonal antibody that blocks LDLR binding to PCSK9. PCSK9 or HIT01 probes were loaded onto Octet sensors and allowed to bind with the AMG145 antibody. The AMG145-bound probes were then used to assess serum samples. As shown in Figure 2B, response levels of PCSK9 and HIT01 immunization groups in the presence of AMG145 were much lower than those in the absence of AMG145 (from 1 to 0.5 nm for the PCSK9 group; from 1.8 to 1.1 nm for the HIT01 group). These results suggested that the immunization elicited responses targeted the AMG145-binding surface—and thus likely the LDLR-binding surface on PCSK9.

To examine the effect of immunizations on the blood cholesterol and lipoprotein levels in mice, we measured the concentrations of LDL, high-density lipoprotein (HDL), and cholesterol in blood samples collected 2 weeks after the second immunization (Figure 2C). Compared to the PBS group, there were significant reductions in LDL, HDL, and total cholesterol concentrations in PCSK9- and HIT01-immunized groups. The HIT01-immunized animals had the largest reduction in LDL concentration (*p* < 0.0001), HDL concentration (*p* < 0.0001), and total cholesterol concentration (*p* < 0.0001). We analyzed cholesterol levels versus LDLR-blocking antibody (Figure 2D), using responses to PCSK9 and excluding responses to PCSK9 blocking with AMG145 as a surrogate for LDLR-blocking antibody responses. The results indicated a correlation between cholesterol levels and AMG145-competing antibody responses elicited in the mice groups. Taken together, the results demonstrate that vaccination by a PCSK9 mimic in mice can elicit antibody responses targeting the AMG145-binding site on PCSK9 and significantly reduce LDL levels.

### Development of second generation of HIT01 immunogens: Cleavage site removal and characterization of HIT01-K21Q-R218E

**Figure 3.**
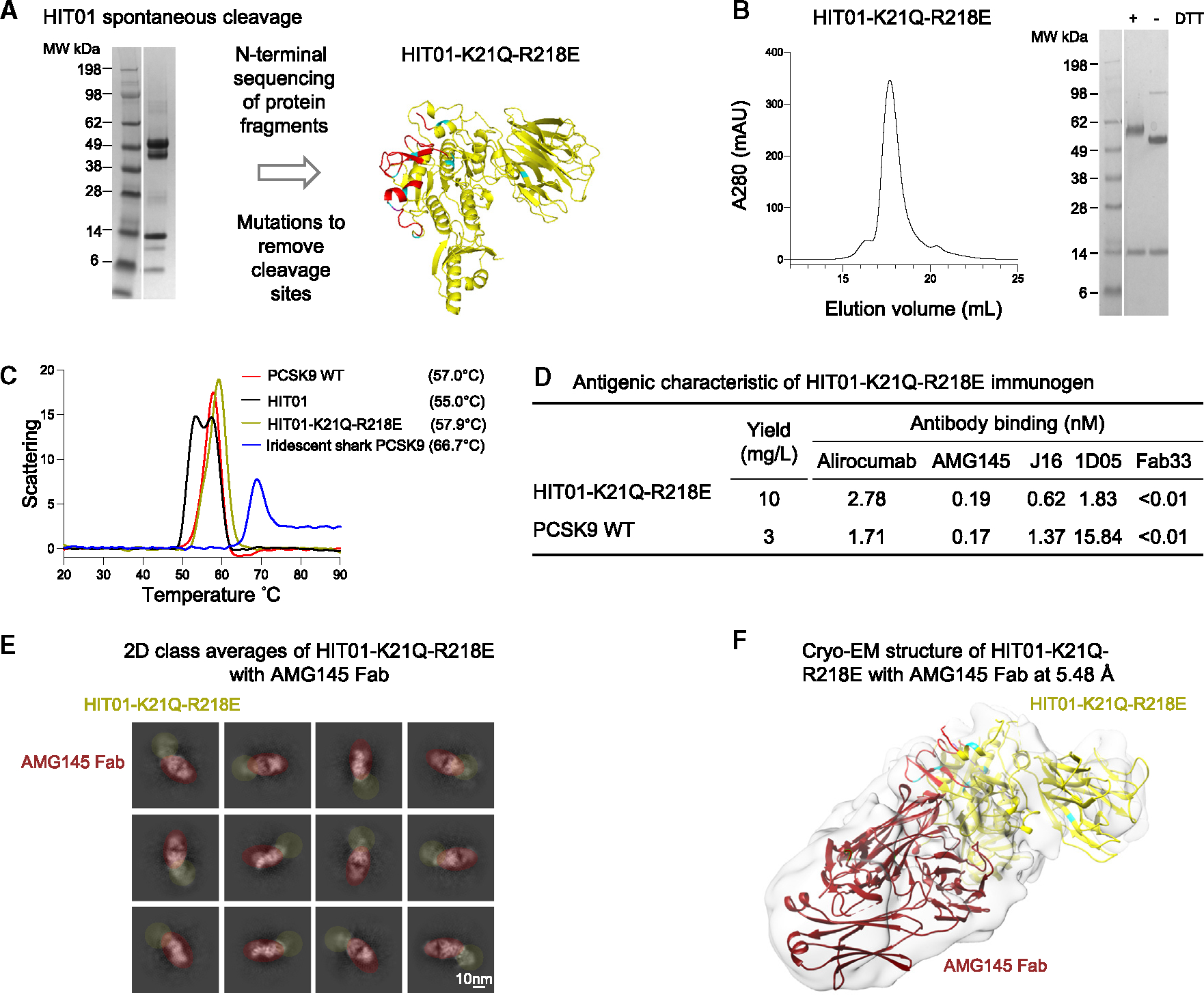
Development of a cleavage-resistant HIT01 immunogen (HIT01-K21Q-R218E) (A) Removal of spontaneous cleavage sites. SDS-PAGE gel image shows cleaved proteins of HIT01 protein stored at 4°C for extended period (more than 1 week). (B) Size-exclusion chromatography and SDS-PAGE gel of HIT01-K21Q-R218E. Ultraviolet trace of HIT01-K21Q-R218E protein purification on size-exclusion chromatograph column (left). SDS-PAGE gel image (right) for HIT01-K21Q-R218 E at reducing and non-reducing conditions. (C) Nano-differential scanning fluorimetry measurements of melting temperature human PCSK9, HIT01, HIT01-K21Q-R218E, and iridescent shark PCSK9. (D) Antigenic characterization and protein expression yields of HIT01-R21Q-R218E immunogen. Binding affinities for HIT01-K21Q-R218E and human PCSK9 to a panel of anti-PCSK9 monoclonal antibodies are shown. (E) 2D class averages of HIT01-K21Q-R218E with AMG145 Fab. HIT01-K21Q-R218E and AMG145 Fab parts are circled by yellow and dark red, respectively. (F) Cryo-EM structure of the HIT01-K21Q-R218E with AMG145 Fab at 5.48 Å. The atomic model is modified from PDB: 3H42. Mutations described in Figure 1 are colored by cyan. See also Figures S2 and S3 and Table S2.

PCSK9 and HIT01 immunogens were produced in HEK923 cell culture through transient transfection. We observed spontaneous cleavage in purified HIT01 after being stored at 4°C for more than 1 week (Figure 3A). To identify cleavage sites, we analyzed SDS-PAGE bands by mass spectrometry and N-terminal sequencing (Figure S2A). We identified two non-native cleavage sites at positions K21 and R218 (Figure S2B) and made mutations K21Q and R218E to remove these two cleavage sites. The cleavage-resistant mutant, HIT01-K21Q-R218E, behaved well on a size-exclusion column and eluted as a single major peak. No spontaneous protein cleavage was observed when the protein was analyzed with SDS-PAGE gel under the same storage condition as HIT01 (Figure 3B). HIT01-K21Q-R218E was evaluated for its thermostability relative to HIT01, human PCSK9, and iridescent shark PCSK9 by nano-differential scanning fluorimetry. Human PCSK9 and HIT01-K21Q-R218E had similar melting temperatures of 57.0°C and 57.9°C, respectively. Iridescent shark PCSK9 had a higher melting temperature of 66.7°C. HIT01 had a split peak that may reflects degradation due to its observed spontaneous cleavage. Moreover, we measured the antigenic property of HIT01-K21Q-R218E together with human PCSK9 against a panel of well-characterized LDLR-blocking antibodies. As shown in Figure 3D, 4 of 5 antibodies recognized the two molecules at similar levels of binding kinetics (less than 3-fold difference) except for 1D05, which bound to HIT01-K21Q-R218E at more than 8-fold higher affinity than to human PCSK9. We also used cryoelectron microscopy (cryo-EM) to examine the HIT01-K21Q-R218E bound to the antigen-binding fragment (Fab) of AMG145 (Figure S3; Table S2). From 2D class averages, structural details could be distinguished for the AMG145 Fab; in contrast, the density for HIT01-K21Q-R218E was smeared, suggesting flexibility (Figure 3E). Thus, while the antibody and PCSK9 mimic were clearly distinguished in the resultant reconstruction density, the flexibility between HIT01-K21Q-R21E domains limited the final resolution at 5.5 Å (Figure 3F).

Overall, these results show that K21Q-R218E mutations reduced the spontaneous protein cleavage of HIT01, stabilizing the HIT01-K21Q-R218E mutant for long-term storage. Further, HIT01-K21Q-R218E behaved well in stability and antigenicity tests, and we explored its use as an improved PCSK9 mimic in vaccine studies.

### NHP immunization with HIT01-K21Q-R218E elicits high-titer AMG145-competing responses and transient reduction of LDL and cholesterol

To assess the vaccine impact of HIT01-K21Q-R218E in NHPs, seven cynomolgus macaques (Table S3) were injected with 100 μg HIT01-K21Q-R218E formulated with Adjuplex^26^ adjuvant per dose and boosted with the same immunogen at 4, 12, and 20 weeks after the first immunization. Blood samples were collected to monitor antibody responses and cholesterol concentrations.

**Figure 4.**
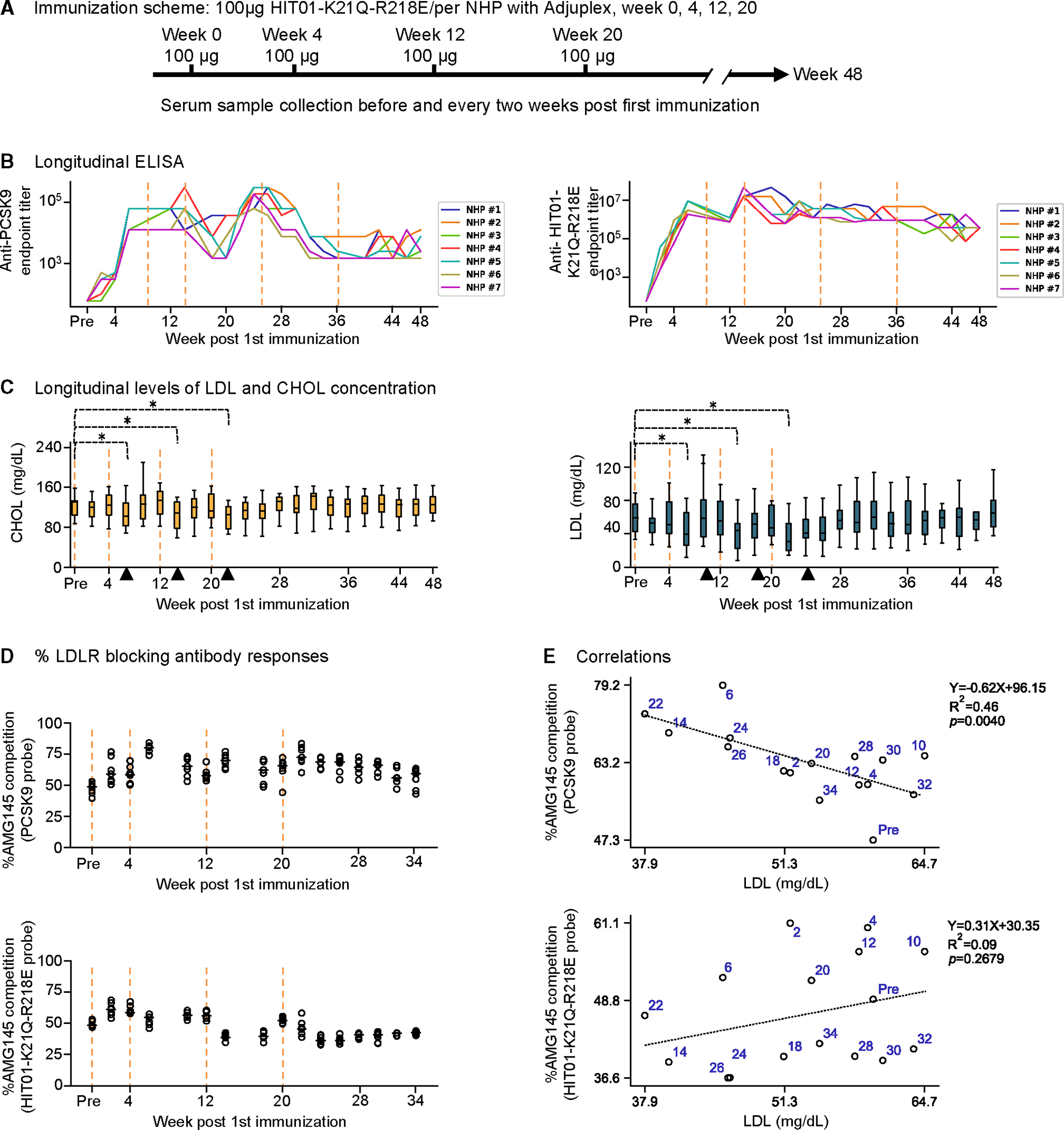
NHP immunization with HIT01-K21Q-R218E elicits AMG145-competing responses and transient reduction of LDL and CHOL (A) HIT01-K21Q-R218E immunization scheme in cynomolgus monkeys. (B) Longitudinal antibody ELISA titers against PCSK9 and HIT01-K21Q-R218E immunogen, respectively. Each line represents one animal. Dotted vertical linesindicate the time points of immunization. (C) Longitudinal LDL and CHOL levels are shown as boxes and whisker plots. Immunization weeks are highlighted; median lines are shown. Significant differences between pre-bleed weeks versus 2 weeks following each immunization were quantified using Wilcoxon signed-rank test, where *p ≤ 0.05. Vertical lines indicate the time points of immunization. (D) AMG145 antibody competition responses (LDLR-blocking antibody responses). Median lines are shown. Vertical lines indicate the time points of immunization. (E) Spearman correlations highlighting the relationships between sera antibody response against the PCSK9-LDLR interaction site (on different probes) versus LDL levels. Values are averaged (mean) over all animals at each selected time point (weeks are shown in blue). p values, linear fit equation, and R^2^ values are also shown. See also Figure S5 and Tables S3 and S4.

Anti-PCSK9 and anti-HIT01-K21Q-R218E ELISA titers in NHP sera showed that immunization with HIT01-K21Q-R218E elicited antibodies against both PCSK9 and HIT01-K21Q-R218E (Figure 4B). The elicited antibodies reacted to cynomolgus PCSK9 at a level similar to that of human PCSK9 (Figure S5). The antibody response was 2–4 orders of magnitude higher for HIT01-K21Q-R218E (622,896 ± 15 endpoint titer geometric mean ± standard deviation over all weeks and NHPs) compared with PCSK9 (6,341 ± 9 endpoint titer geometric mean ± standard deviation over all weeks and NHPs). Fluctuations were observed in anti-PCSK9 titers, with anti-PCSK9 titers falling an order of magnitude at weeks 18 and 20, rising to peak levels at weeks 24 and 26, and then falling two orders of magnitude by week 32, while anti-HIT01-K21Q-R218E titers also fluctuated but to a lower extent. Interestingly, a relatively strong correlation was observed between circulating PCSK9 levels (as discussed below) and anti-PCSK9 titers (r(8) = 0.81, *p* = 0.005; mean over all NHPs at a time point).

Next, we measured the impact of the elicited antibody responses on circulating LDL and cholesterol levels (Figure 4C; Table S4). Circulating LDL and cholesterol levels were, on average, 55 ± 26 and 119 ± 29 mg/dL (mean ± SEM), respectively, over all NHPs and time points (Figure 4C). LDL and cholesterol levels were highly correlated (r(20) = 0.86, *p* < 0.001; mean over all NHPs). A significant reduction in LDL and cholesterol (compared with pre-immunization levels) was observed 2 weeks after each immunization: weeks 6 (Z = 2.15, *p* = 0.031; Z = 2.42, *p* = 0.016), 14 (Z = 2.42, *p* = 0.016; Z = 2.42, *p* = 0.016), and 22 (Z = 2.42, *p* = 0.016; Z = 2.42, *p* = 0.016), respectively. LDL and cholesterol returned to pre-immunization levels after 2 weeks in all cases. These results suggest that HIT01-based immunization was effective but its impact was transient.

To assess on-target antibody elicitation, we used AMG145 to quantify the proportion of elicited antibodies that block PCSK9 interaction with the LDLR (Figure 4D). Between 40% and 84% (mean over all weeks and NHPs of 63% ± 10%) of the elicited wild-type (WT) PCSK9-binding antibody responses were competed by AMG145, indicating that about 40%–80% of elicited antibodies could block LDLR binding on WT PCSK9. Compared with anti-WT PCSK9 responses, a lower proportion of the elicited antibody responses against HIT01-K21Q-R218E were competed by AMG145 (between 33% and 68%, mean over all weeks and animals of 47% ± 9%), although substantially higher antibody responses against HIT01-K21Q-R218E than against WT PCSK9 were observed (Figure 4B). These results suggest that immunization of NHPs with HIT01-K21Q-R218E could elicit substantial antibody responses against PCSK9, with a significant proportion being directed toward the LDLR-binding site.

As expected, an inverse correlation was observed between LDL levels and the percentage of elicited antibody responses that could be competed by AMG145, which blocks PCSK9 binding to LDLR (Figure 4E) (r(14) = −0.64, *p* < 0.007; mean over all NHPs at a time point). This observation supported the accuracy and precision of sera profiling (Figure 4E). Interestingly, for the LDLR site on HIT01-K21Q-R218E, no significant correlation was observed between LDL levels versus the proportion of elicited LDLR-directed antibodies, suggesting the proportion of the response directed at the LDLR site on HIT01 alone to not be predictive of LDL reduction.

Overall, these results suggested that the immunization elicited antibody responses that were responsible for the LDL reduction observed, although the effect was transient.

### The presence of PCSK9-reactive antibodies alters PCSK9 serum half-life, increases PCSK9 levels up to 10-fold, and confounds HIT01 vaccine efficacy

**Figure 5.**
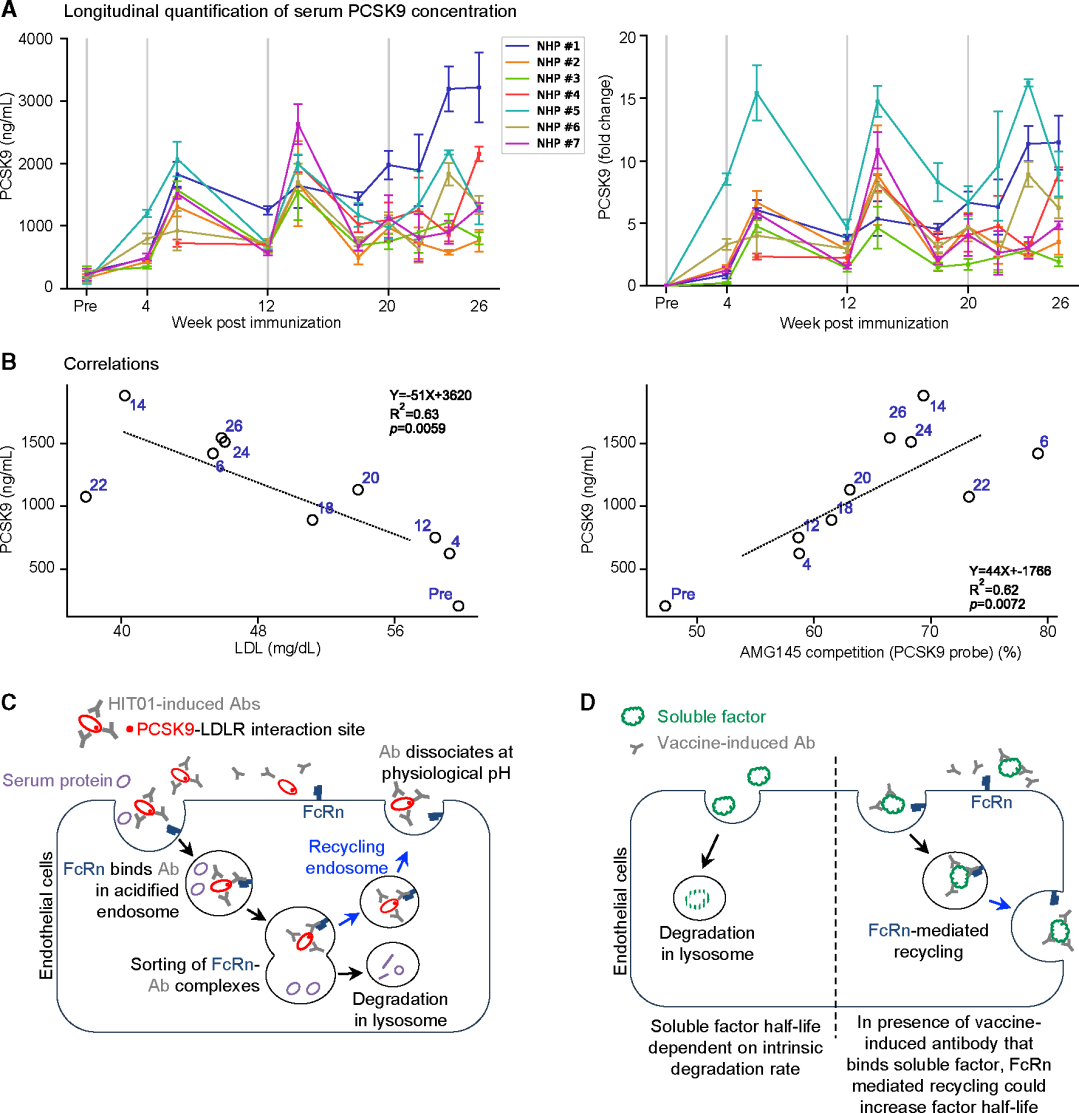
The presence of PCSK9 antibodies alters PCSK9 serum-half life, confounding vaccine efficacy (A) Longitudinal serum PCSK9 concentrations for each NHP are shown on the left and the fold change in concentration from baseline on the right. For immunization weeks, all replicate measurements for each week were averaged. For pre-bleed weeks, all replicate measurements for several weeks were averaged. Vertical lines indicate the time points of immunization. For fold change measurements, the relative change equals “(PCSK9 measurement – avg pre-bleed)/avg pre-bleed”; the average over pre-bleed weeks was considered the initial value. (B) Spearman correlations highlighting unanticipated relationships between PCSK9 levels, LDL levels, and sera antibody response against the PCSK9-LDLR interaction site. Values are averaged (mean) over all animals at each selected time point (weeks are shown in blue). p values, linear fit equation, and R^2^ values are also shown. (C) Schematic of antibody/FcRn-mediated PCSK9 recycling and hypothesized mechanism of PCSK9 increase. (D) Schematic of antibody/FcRn-mediated pathway for half-life extension in which soluble factors enter the recycling endosome through antibody-medicated interactions with FcRn versus being directed to the lysosome for degradation. See also Table S5.

To better understand the relationship between circulating PCSK9 levels and LDL levels post-HIT01-K21Q-R218E immunization, immunized NHP sera were assessed for PCSK9 concentrations. PCSK9 levels followed a similar up-down trend across all participants until around week 20 (Figure 5A; Table S5). Over the first 20 weeks, PCSK9 levels ranged from 336 to 2,629 ng/mL, with a mean of 1,129 ± 543 ng/mL. Closer examination showed most NHPs to have experienced a substantial increase in PCSK9 levels compared to pre-immunization levels (~5- to 10-fold increase), with NHP #5 experiencing the largest increase (~15-fold). These results indicate PCSK9 to accumulate to relatively high concentrations in the bloodstream post immunization.

Circulating LDL levels and elicited antibody levels were then compared with PCSK9 levels. A significant and unexpected correlation (inverse relationship) was observed between PCSK9 levels versus LDL levels (r(8) = ~0.77, *p* = 0.009; mean over all NHPs at a time point) (Figure 5B, left). Thus, unlike PCSK9 levels versus LDL levels in unvaccinated subjects, where the level of PCSK9 correlates with LDL level, in the vaccinated NHPs, the opposite was observed. An explanation for this seeming paradoxical correlation is that in vaccinated animals, there are two competing effects: (1) the elicitation of PCSK9-binding antibodies that recognize the LDLR-binding site on PCSK9, blocking PCSK9-induced degradation of the LDLR and enabling the LDLR to recycle and degrade LDL to reduce LDL levels, and (2) the elicitation of PCSK9-binding antibodies that increase the half-life of PCSK9, thereby increasing the level of PCSK9 in serum. The resulting outcome of these two competing effects depends on the degree to which the PCSK9-binding antibody inhibits PCSK9 binding to the LDLR—with the observed inverse correlation indicating that the first effect, the elicited antibody blocking PCSK9-induced degradation of the LDLR, appears to dominate. Relevant to this, we observed a significant correlation (direct relationship) between PCSK9 levels versus LDLR-directed antibody levels (WT PCSK9 as probe) (r(8) = 0.75, *p* = 0.013; mean over all NHPs at a time point), showing AMG145 competition to increase as PCSK9 levels increased. Thus, vaccine-induced PCSK9-binding antibodies caused the circulating PCSK9 levels to increase and uncoupled the circulating PCSK9 level from the LDL level, as the antibody-bound PCSK9 was incapable of raising LDL levels in the bloodstream. This is consistent with the reported observation in clinic that in patients injected with anti-PCSK9 antibodies (for therapeutic purposes), bloodstream PCSK9 levels increase up to 20-fold from baseline.^27–29^

A model of the hypothesized mechanism of transient LDL reduction (via immunization with HIT01-K21Q-R218E) is shown in Figure 5C. Opposing effects from vaccine-elicited antibodies support an immunization-based feedback loop, where the total PCSK9 levels increase as the functional PCSK9 levels decrease over the immunization regimen.

## DISCUSSION

Immunization with self-immunogens poses multiples challenges including self-reactivity and breakthrough immunogen tolerance. In this study, the binding regions for the LDLR and therapeutic antibodies were transplanted from human PCSK9 to a homologous scaffold derived from the iridescent shark (*Pangasianodon hypophthalmus*). Antigenic analysis indicated transplantation to have preserved the integrity of the transplanted binding surface on the scaffold molecule, HIT01. This PCSK9-mimicking immunogen elicits high-titer antibody responses against itself and, to a lesser extent, against PCSK9 in both murine and NHP models. A portion of the elicited antibodies competed with AMG145, a monoclonal antibody that has been approved for the treatment of high cholesterol in humans. At 2 weeks after vaccination, significant reductions of LDL were observed in both murine and NHP models. The data indicate that vaccine-elicited antibodies behave the same way as therapeutic monoclonal antibodies in terms of blocking PCSK9 binding to the LDLR. However, we observed the levels of PCSK9 to increase up to 5-fold. The increased level of PCSK9 could have resulted from several factors, including a change of the homeostatic level of PCSK9 or the PCSK9-binding antibody altering PCSK9 recycling, increasing the half-life of PCSK9 and leading to an accumulation of PCSK9. Because PCSK9 has a half-life in plasma of approximately 5 min under normal conditions,^30–32^ and serum antibodies have half-lives of ~21 days,^33,34^ much longer than that of PCSK9, the second explanation likely dominates, as illustrated in Figure 5C, with the antibody extending the half-life of PCSK9, leading to increased concentrations of PCSK9 in blood.

Increased concentrations of PCSK9 likely diminish the vaccine-induced blocking of the PCSK9-LDLR interaction and provide an explanation for the somewhat paradoxical results of Figure 5B (left), where increased concentrations of PCSK9 in vaccinated macaques led to lower LDL levels.

Overall, our interpretation of these results is consistent with the observed transient decrease of LDL cholesterol 2 weeks after immunization in NHPs, with the increased levels of PCSK9 likely resulting in a rebound in the level of LDL and cholesterol. Consistent with our results, increased PCSK9 levels have also been observed in the passive administration of PCSK9 monoclonal antibodies and PCSK9 vaccine studies.^22,27,29,35–40^ Because antibodies elicited by HIT01 likely target multiple sites on PCSK9—not just the LDLR-binding surface—the vaccine-elicited antibody-induced increase in the half-life of PCSK9 may be greater than the increase from the passive administration of antibodies that solely target the LDLR-binding site.

Thus, immunization with a PCSK9-mimicking immunogen indeed elicited antibody responses that decreased LDL levels upon vaccination. The elicited polyclonal antibodies competed with PCSK9-targeting monoclonal antibodies, as assessed clinically. The PCSK9-mimicking immunogens could be improved if the immune response were to focus more specifically on PCSK9 surfaces that interact with the LDLR. Such altered focusing might be achieved through glycan masking of non-desired epitopes.^41–43^

Lastly, it has not escaped our attention that our results suggest a means for increasing the half-life of soluble regulatory factors by vaccination. For example, the appetite-sensing hormone leptin has a half-life in the range of 40 min to 1.5 h.^44,45^ A vaccine targeting non-functional surfaces of leptin should elicit antibodies that bind to this soluble factor, altering its recycling and increasing its half-life, a consequence of potential utility.^44,46,47^ Thus, the observed extended half-life of vaccine targets through FcRn recycling may provide a general means to improve the soluble factor half-life (Figure 5D).

### Limitations of the study

We observed a transient LDL reduction 2 weeks after a second, third, or fourth immunization of the PCSK9 mimic, followed by an increased level of PCSK9 and a flat LDL concentration. The dramatic increase in total PCSK9 in blood upon vaccination appears to limit the efficacy of HIT01-K21Q-R218E as a vaccine immunogen to reduce LDL. There are several ways to improve the immunogen to elicit antibody responses that are more focused on blocking LDLR binding and might thereby minimize the increase of PCSK9 concentration. One approach is to mask the surface of the HIT01-K21Q-R218E protein with glycans exposing only the LDLR-binding surface.

In addition to a reduction in LDL and cholesterol, we also observed a reduction of HDL levels in mice at 2 weeks post-second immunization. The mechanism for this reduction in HDL levels is unclear and may warrant further investigation.

## STAR★METHODS

Detailed methods are provided in the online version of this paper and include the following:

### RESOURCE AVAILABILITY

#### Lead contact

Further information and requests for resources and reagents should be directed to and will be fulfilled by Peter D. Kwong (pdkwong@nih.gov).

#### Materials availability

Plasmids generated in this study are available upon request.

#### Data and code availability

- Cryo-EM map has been deposited with EMDB as entry ID EMD-41409.
- This paper does not report original code.
- Any additional information required to reanalyze the data reported in this paper is available from the Lead Contact upon request.

### EXPERIMENTAL MODEL AND SUBJECT DETAILS

#### Animal models

Mouse and NHP experiments were carried out in compliance with National Institutes of Health regulations and approval from the Animal Care and Use Committee of the Vaccine Research Center. Animals were housed and cared for in accordance with local, state, federal and institutional policies in facilities accredited by AAALAC International under standards established in the Animal Welfare Act and the Guide for the Care and Use of Laboratory Animals. BALB/cJ mice, six-week-old female, were purchased from Jackson Laboratories. Indian rhesus macaques of both sexes, age 6–7 years, without prior involvement in procedures or drug experimentation were used for the NHP immunization studies.

#### Cell lines

Expi293F cells were purchased from ThermoFisher Scientific Inc. Cells were maintained in Expi293 Expression Medium. The cell line was used directly from the commercial sources following manufacturer suggestions as described in Method details below.

### METHOD DETAILS

#### Immunogen design

We analyzed the structures of human PCSK9 in complex with LDL-receptor (PDB: 3P5B) as well as with monoclonal antibodies reported to reduce cholesterol levels when passively infused (PDB: 3H42, 3SQO, 5VL7, 2XTJ). We identified PCSK9 residues that contacted either LDLR or any of the four monoclonal antibodies that have been reported to reduced cholesterol levels. Seven stretches of contacting residues were identified contacting at least two of these PCSK9 binding ligands (LDLR or the four monoclonal antibodies). The seven stretches of peptide were then transplanted onto PCSK9 homologs. We then searched the resultant transplants for 9-mer peptide in common with known human proteins and introduced point mutations that disrupt 9-mer stretches in common with human proteins.

#### Antigenicity screening of designed PCSK9 mimics

In each well of a 96-well cell culture microplate (Corning Scientific, NY), 2.5 × 10^4^ log-phase HEK 293T cells in 100 μL of RealFect Expression medium (ABI Scientific, VA) were allowed to grow for 24 h at 37°C, 5% CO_2_. Immediately before transfection, 40 μL of spent medium per well was removed. 250 ng of plasmid DNA encoding a PCSK9 immunogen variant with a HIS tag in 10 μL of Opti-MEM Reduced Serum medium (Thermo Fisher Scientific, CA) was mixed with 0.75 μL of TrueFect Max transfection reagent (United Biosystems, MD) and with 10μL of Opti-MEM Reduced Serum medium at room temperature (RT) for 15 min, then combined with growing cells per well in the 96-well cell culture microplate. Transfected cells were incubated at 37°C and 5% CO_2_ for overnight (about 15 h), and then fed with 25 μL per well of CelBooster medium (Cell Growth Enghancer for adherent cells, ABI Scientific) with additional 3x Streptomycin-Penicillin and 10% FBS. Four days post transfection, supernatants from each cell well were harvested and analyzed in a 96-well plate formatted ELISA. Briefly, 30 μL of expression supernatant and 70 μL of PBS per well were incubated in Ni-coated plate (Thermo Scientific) at RT for 2 h. Captured PCSK9 immunogens were detected by incubating with anti-PCSK9 primary antibodies (human IgG) at 10 μg/ml concentration at RT for 60 min, followed with a secondary anti-human IgG Fc HRP-conjugate (Jackson ImmunoResearch Labs) at RT for 30 min. After final washing, the reaction signal was developed with tetramethylbenzidine substrate (BioFX-TMB, SurModics, MN) at RT for 10 min. The reaction was stopped by the addition of 0.5 N H_2_SO_4_. The signal was measured at 450 nm on a micro plate reader (SpectraMax Plus, Molecular Devices, CA). All samples were measured in duplicate.

#### Protein expression and purification

Genes encoding PCSK9 and variant proteins were codon-optimized, synthesized and subcloned (Gene Universal Inc, Newark, DE) into pVRC8400 (CMV/R) expression vectors. For protein expression, 3 mL of Turbo293 transfection reagent (Speed BioSystems) was mixed with 50 mL Opti-MEM medium (Life Technology) and incubated at room temperature (RT) for 5 min 1 mg plasmid DNAs was mixed with 50 mL of Opti-MEM medium in a separate tube, and the mixture added to the Turbo293 Opti-MEM mixture. The transfection mixture was incubated for 15 min at RT then added to 800 mL of Expi293 cells (Life Technology) at 2.5 million cells/ml. The transfected cells were incubated overnight in a shaker incubator at 9% CO_2_, 37°C, and 120 rpm. On the second day, about 100 mL of Expi293 expression medium was added. On day 5 post transfection, supernatants were harvested, filtered. Proteins were purified from the supernatant using Ni-NTA and strep chromatography. Proteins were further purified by size exclusion chromatography on Superdex 200 Increase 10/300 GL in PBS buffer.

#### Monoclonal antibody expression and purification

Variable regions for AMG145, Fab33 etc were codon optimized, synthesized and subcloned (Gene Universal Inc, Newark, DE) into pVRC8400 (CMV/R) expression vectors containing human IgG constant regions. A heavy chain plasmid containing an HRV3C cleavage site in the hinge region was used for Fab production. Corresponding heavy and light chain plasmids were co-transfected in Expi293F cells using Turbo293 transfection reagent.^57^ Briefly, 0.75 mL of Turbo293 transfection reagent (Speed BioSystems) were added to 12.5 mL Opti-MEM medium (Life Technology) and incubated for 5 min at room temperature. Meanwhile, 125 μg of heavy chain and 125 μg of light chain plasmid DNA were added to 12.5 mL of Opti-MEM medium in another tube. The Opti-MEM medium containing Turbo293 were then added to plasmid DNAs, incubated for 15 min at room temperature, and added to 200 mL of Expi293 cells (Life Technology) at 2.5 million cells/ml. The transfected cells were cultured in shaker incubator at 120 rpm, 37°C, 9% CO_2_. Culture supernatant was collected 5 days after transfection and loaded onto a protein column. After washing the columns with PBS, IgG was eluted using a low pH buffer and immediately neutralized with 10% 1 M Tris-Cl, pH 8.0. To produce antibody Fab, purified IgG was cleaved by HRV3C protease and the digest was passed over a protein A column to separate the Fab fragments. The Fab fragments were purified further by size-exclusion chromatography using a Superdex 200 column in PBS buffer.

#### NHP serum antibody responses analysis (ELISA)

Anti-HIT01 and anti-PCSK9 ELISA were performed using ninety-six well Costar half plates (Costar High Binding Half-Area; Corning, Kennebunk, ME) that were coated (50 μL/well) with 2 μg/mL of either HIT01 K21Q R218E or Human PCSK9 CHS overnight at 4°C. The following morning, they were washed five times with PBS-T (0.05% tween/PBS) and then blocked with 5% skim milk/PBS blocking buffer (100 μL/well) for 1 h at room temperature. The plates were then washed fix times with PBS-T. Serially diluted NHP plasma starting at a 1:20 or 1:1000 dilution was then additionally added to the wells for 1 h at room temperature. The plates were then washed five times with PBS-T. HRP-conjugated goat anti-monkey IgG secondary antibodies were added to the plate at 1:5000 dilution for 1 h at room temperature. The plates were then washed five times with PBS-T. The plates were then developed with tetramethylbenzidine (TMB) (50 μL/well) substrate for 10 min at room temperature in the dark. The reaction was stopped by adding 1N sulfuric acid (50 μL/well) and the optical density (OD) of each well was read at 450 nm.

#### Serum cholesterol concentration determination

Cholesterol concentration in serum samples collected from mouse and NHP studies were determined at Charles River Laboratory on a Beckman Coulter AU5800 Clinical Chemistry instrument. Concentrations of low-density lipoprotein, high-density lipoprotein, and Cholesterol were determined following the manufacturer’s protocol.

#### Biolayer interferometry assay

A fortéBio Octet Red384 instrument was used to measure sera recognition of human PCSK9 and HIT01 targeting antibodies. A series of scouting experiments at different sera dilutions, buffer conditions and sensor tips were performed to minimize non-specific binding. Optimum non-specific binding was obtained using HIS1K sensor tips and sera dilutions in the range of 1:200 in PBS supplemented with 1% BSA. Assays were performed at 30°C in tilted black 384-well plates (Geiger Bio-One) with agitation set to 1,000 rpm in PBS supplemented with 1% BSA and a well volume of 50 μL. All experiments were performed in duplicate. Variability in loading was within a row of eight tips did not exceed 0.1 nm for each of these steps. Parallel correction to subtract systematic baseline drift was carried out by subtracting the measurements recorded for a loaded sensor incubated in PBS +1% BSA. The association after 300 s was recorded. For AMG145 antibody blocking assays, 50 mg/ml of AMG145 IgG was used to block PCSK9 or HIT01 probe-loaded sensors. The antibody blocked human PCSK9 or HIT01 loaded sensors were then used to probe antibodies in serum samples. Data analysis was performed using Octet and GraphPad Prism 6 software.

For monoclonal antibody binding kinetics analysis, all assays were carried out with agitation set to 1,000 rpm in 1x Kinetics Buffer (fortéBio) using solid black 384-well plates (Geiger Bio-One). Briefly, PCSK9 or variant proteins at 50 μg/mL in PBS were loaded onto Ni-NTA biosensors using their C-terminal histidine tags for 300s. Biosensor tips were then equilibrated for 60 s in 1x Kinetics Buffer prior to assessment of binding to the monoclonal antibody Fab molecules in solution (0.00625–0.4 μM). Association was allowed to proceed for 300 s followed by dissociation for 600 s. Dissociation wells were used only once to prevent contamination. Parallel correction to subtract systematic baseline drift was carried out by subtracting the measurements recorded for a sensor loaded with PCSK9 or variant molecules incubated in 1x Kinetics Buffer.

#### Serum PCSK9 concentration measurement

NHP PCSK9 levels were determined via ELISA using the Human Proprotein Convertase 9/PCSK9 Quantikine ELISA Kit (R&D Systems, cat. no. SPC900), according to the manufacturer’s instructions. Non-heat-inactivated NHP serum was serially diluted starting at 1:20. Concentrations were calculated by taking the average of the duplicates.

#### Mass spec N-terminal amino acid sequence determination

Protein samples were separated on SDS-PAGE gel and transferred to PVDF membrane. Band of each protein was cut off from PVDF membrane and was analyzed at Creative Proteomics (Shirley, NY) on an ABI Procise 494HT (Thermo Fisher). The procedure determines the N-terminal amino acid sequence of proteins and peptides by the Edman degradation chemistry.

#### Differential scanning fluorimetry

Melting temperatures of the PCSK9 and HIT01-K21Q-R218E proteins were determined by nano differential scanning fluorimetry (nanoDSF) on a Prometheus NT.48 instrument (NanoTemper Technologies). A total of 10 μL of protein samples at a concentration between 1.0 mg/mL in PBS were loaded into capillaries and placed on the sample holder. A temperature gradient from 20°C to 90°C was scanned at 1°C/min, and the intrinsic fluorescence intensity ratio (350 nm:330 nm) was recorded. The data analysis was performed using Prometheus NT.48 control software.

#### Animal immunization

Three groups (10 animals each group) of six-week-old female BALB/cJ mice (Jackson Laboratories) were inoculated intramuscularly with 10ug human PCSK9 or HIT01 protein with 1: 10 ratio of adjuvant Alum (Vaccine Production Program, VRC, Lot# D-15-0017) as adjuvant at weeks 0 and 2. Serum was collected 2 weeks post the second immunization for measurements of antibody responses and cholesterol. Seven NHP were immunized with 100ug HIT01-K21Q-R218E per animal with 20% Adjuplex (Empirion LLC, Columbus, OH) as adjuvant at week 0, 4, 12, 20. Serum samples were collected at 2 week interval from 150 days before the first immunization through 365 days post the first immunization (Table S3).

#### Electron microscopy

For cryo-grids preparation, aliquots (4 μL) of samples were applied to glow-discharged C-flat Au grids (R1.2/1.3, 400 mesh) inside the chamber of an FEI Vitrobot IV (4°C and 100% humidity). Grids were flash frozen in liquid ethane and using an FEI Glacios microscope operated at 200 kV for data collection. Images were collected using a GIF K3 camera (Gatan) with SerialEM^56^ in the super-resolution counting and movie mode, at a nominal magnification of 45,000×, which renders a final pixel size of 0.46 Å at object scale (superresolution), and with defocus ranging from −1 to −2.5 μm. A total of 40 frames were collected for each micrograph stack. The dose rate was 20 e/pixel/s.

#### Image processing

Collected micrographs were manually screened. A total of 4801 qualified movie stacks were selected for image processing (Figure S3). Data process workflow, including motion correction, CTF estimation, particle picking and extraction, 2D classification, ab-initio reconstruction, homogeneous refinement, heterogeneous refinement, and non-uniform refinement were carried out in cryoSPARC 3.3.^51^ The overall resolution of HIT01-K21Q-R218E with AMG145 Fab density map is 5.48 Å (328 K particles). The coordinate of the human PCSK9 with mAb1 (the precursor of AMG145) in PDB: 3H42 was used as initial model for fitting the cryo-EM map. The resolution estimation was based on gold-standard FSC at the cutoff of 0.143. Structural analysis was done with UCSF Chimera 1.11.2^48^ and Pymol (http://pymol.org).

### QUANTIFICATION AND STATISTICAL ANALYSIS

Statistical differences for BLI antibody responses and for cholesterol levels between different mouse groups were determined by performing un-paired non-parametric two-tailed Mann–Whitney tests using the GraphPad Prism version9. For longitudinal NHP cholesterol significant differences were quantified using the Wilcoxon signed-rank test, where *p*-value *≤ 0.05, **≤ 0.005, ***≤ 0.0005. For correlational analysis, Spearman’s rank correlation measure was performed using the SciPy spearman module (default settings, uncorrected for multiple comparisons). Prior to rank correlation quantification, measurements were averaged over all subjects within a group.

## Supplementary Material

1

## Figures and Tables

**KEY RESOURCES TABLE:** 

REAGENT or RESOURCE	SOURCE	IDENTIFIER

Antibodies		

J16	PDB: 3SQO	3SQO
mAb1	PDB: 3H42	3H42
Fab33	PDB: 5VL7	5VL7
1D05	PDB: 2XTJ	2XTJ
PCSK9-LDLR	PDB: 3P5B	3P5B
Evolucumab (AMG145)	GenomeNet Database Resources https://www.genome.jp/	D10557
Alirocumab	GenomeNet Database Resources https://www.genome.jp/	D10335

Chemicals, Peptides, Recombinant Proteins and Biosensors		

Pierce Protein A Agarose	ThermoFisher Scientific	20334
Octet Anti-Penta-HIS (HIS1K) Biosensors	SARTORIUS	Item No.: 18-5120
Octet Kinetics Buffer 10X	SARTORIUS	Item No.: 18-1105
Octet Ni-NTA (NTA) Biosensors	SARTORIUS	Item No.: 18-5101
Adjuplex	Empirion LLC, Columbus, OH	
Alum adjuvant	Vaccine Production Program, VRC	Lot# D-15-0017
Human Proprotein Convertase 9/PCSK9 Quantikine ELISA Kit	R&D Systems	cat. no. SPC900
Opti-MEM medium	ThermoFisher Scientific	31985070
Turbo293 transfection reagent	SPEED BioSystem	PXX1002
AbBooster medium	ABI scientific	PB2668

Deposited Data		

HIT01-K21Q-R218E with AMG145 Fab map	EMDB	EMD-41409

Experimental Models: Cell Lines		

Expi293F Cells	ThermoFisher Scientific	A14527
Expi293F cells	ThermoFisher Scientific	A14527

Software and Algorithms		

Chimera	Pettersen et al.^48^	https://www.cgl.ucsf.edu/chimera/
ChimeraX	Pettersen et al.^49^	https://www.cgl.ucsf.edu/chimerax/
Coot	Emsley and Cowtan^50^	https://sbgrid.org/software/
CryoSparc	Punjani et al.^51^	https://guide.cryosparc.com/
MolProbity	Barad et al.; Williams et al.^52,53^	http://molprobity.biochem.duke.edu
PDBePISA	Krissinel et al.^54^	https://www.ebi.ac.uk/pdbe/pisa/
Phenix	Adams et al.^55^	https://sbgrid.org/software/
PRISM	GraphPad Software	https://www.graphpad.com/scientific-software/prism/
Pymol	Schrödinger LLC	https://pymol.org
SerialEM 4.0	Mastronarde^56^	https://bio3d.colorado.edu/SerialEM/

## Highlights

- Designed a human PCSK9 mimic with no consecutive 9 residues of any human protein
- Immunization with the PCSK9 mimic significantly reduced cholesterol and LDL levels
- Vaccine-induced antibodies altered PCSK9 half-life, confounding vaccine impact
