# Supplementary material for: Cholesterol reduction by immunization with a PCSK9 mimic
Source: Cell Rep. Author manuscript; Available in PMC 2024 Aug 7. (PMC11305080; doi:10.1016/j.celrep.2024.114285)
Supplement: 1 [file NIHMS2005218-supplement-1.pdf]

Cell Reports, Volume *43*

## Supplemental information

# Cholesterol reduction by immunization with a PCSK9 mimic

**Baoshan Zhang, Gwo-Yu Chuang, Andrea Biju, Daniel Biner, Jiaxuan Cheng, Yiran Wang, Saran Bao, Cara W. Chao, Haotian Lei, Tracy Liu, Alexandra F. Nazzari, Yongping Yang, Tongqing Zhou, Steven J. Chen, Xuejun Chen, Wing-Pui Kong, Li Ou, Danealle K. Parchment, Edward K. Sarfo, HaoMin SiMa, John-Paul Todd, Shuishu Wang, Ruth A. Woodward, Cheng Cheng, Reda Rawi, John R. Mascola, and Peter D. Kwong**

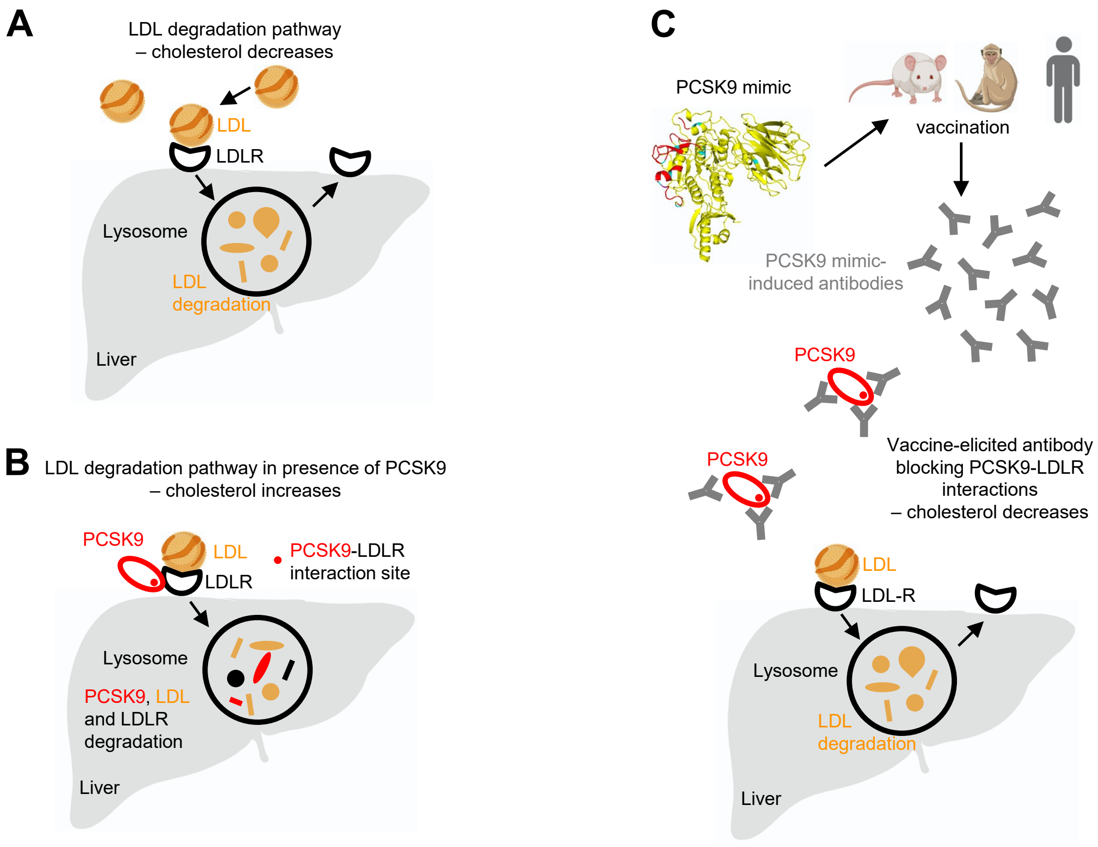


**Figure S1. Schematic for decreasing cholesterol by inducing immune responses to PCSK9 that block PCSK9 interactions with LDLR, related to Figure 1**

(**A**) Cholesterol decreases in LDL degradation pathway with or without presence of PCSK9.

(**B**) PCSK   mimic vaccination induces antibody targeting PCSK9. Antibody-PCSK9 complexes block PCSK9 interactions with LDLR, resulting in decrease in cholesterol.

## A

HIT01 spontaneous cleavage

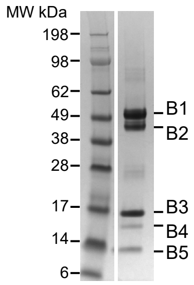


| | Protein band sample | | | | | |
|---|---|---|---|---|---|---|
| | AA# | B1 | B2 | B3 | B4 | B5 |
| Amino acid detected | 1 | (S), (R, D) | (H, S, G) | D | (E, S, H, D, A, G) | S, (T, G) |
| | 2 | I | (A, G) | Y | V | I, (P) |
| | 3 | P | (P, S) | S | K, (S) | P, (F) |
| | 4 | (W, E) | (K) | E | A | (W), (N, Y) |
| | 5 | N | (N) | D | P | N |
| | 6 | L | (E, L) | K | Q | L |
| | 7 | Q | | E | L, (G, R, Q) | Q, (A) |
| | 8 | (R) | | V | (D) | R |
| | 9 | I | | K | (H) | I, (V) |
| | 10 | (I) | | A | (P, G) | I, (T) |
| N terminal position # on PCSK9 | | 130 | 218 | 16 | 21 | 130 |

## B

HIT01 amino acid sequence

Normal processing

Non-specific cut

mhlvlvlclaalaacDYSEDKEVKAPQLDHPDPGTERVAELLRCTKSVWRIPEQYLVVLREGTRDSHVQRTVST
LRAQAARRGHAIHIMHTYSGVFHGFLIKMSSEVLPMALKLPHVAYIEEDSSIFAQSIPWNLQRIIQTKHETGK
YTPPNDGAQVTVFLLDTSVQTDHREIEGKVMVTDFNSMPKEDGTRFHRSASKCESHGTHIAGVLSGRDA
GVARGVSVNTVRVLNCQGRGTVSGALAGLEYIRASLQAQPVSPVIILLPFVGGFSRTLNTACREMVHSGA
VLIAAAGNYQDDACMYSPASEPEVITVGASNAADRPLSSGTTGTNLGRCVDVFAPGEDIISASGDCSTCFV
SMSGTSQSAAHAAGIAAVLLNAYPSASPAEVLQLLRYHAVQRVINPDSLPPEHYLTTPDMVAALPTSAATG
EKLLCRSVWSKRSGVGSFDTAVARCRHGEEMFSCSSYSPNGVHAGERIEIRDGQKVCEAHHGIGGQGV
YAVARCCTGSRVKCHASASLHVGIDAECPSQEFQLTGCSSHYIRSQDVAQPSWPLHSNRKACPAGEGG
TSHAFCCHAPNLECHLIEHHQSEFTKQVEVSCEDSWTLTGCNAVSHGSVTHAAYTRGNTCVIQMFGGDK
GAAAIAICCRYRPLDQQSNNNHEQNT

**Figure S2. Non-specific protease cleavage sites of recombinant HIT01 identified using mass-spec analysis, related to Figure 3**

(**A**) Identification and removal of cleavage of HIT01 to make HIT01-K21Q-R218E. SDS-PAGE gel analysis indicating unexpected protease cleavage of HIT01 protein. Bands B1 and B3 are the same bands observed in wild type PCSK9 proteins. Band B2, B4, and B5 are extra bands due to protease cleavage. Mass-spec analysis results for each of the 5 bands are listed in the table.

(**B**) Amino acid sequence of HIT01 molecule with arrows showing native and unexpected protease cleavage sites. Arrows indicate sites during natural PCSK9 processing (green) and no-specific cleavage (brown). Signal peptide sequence is indicated in low case.

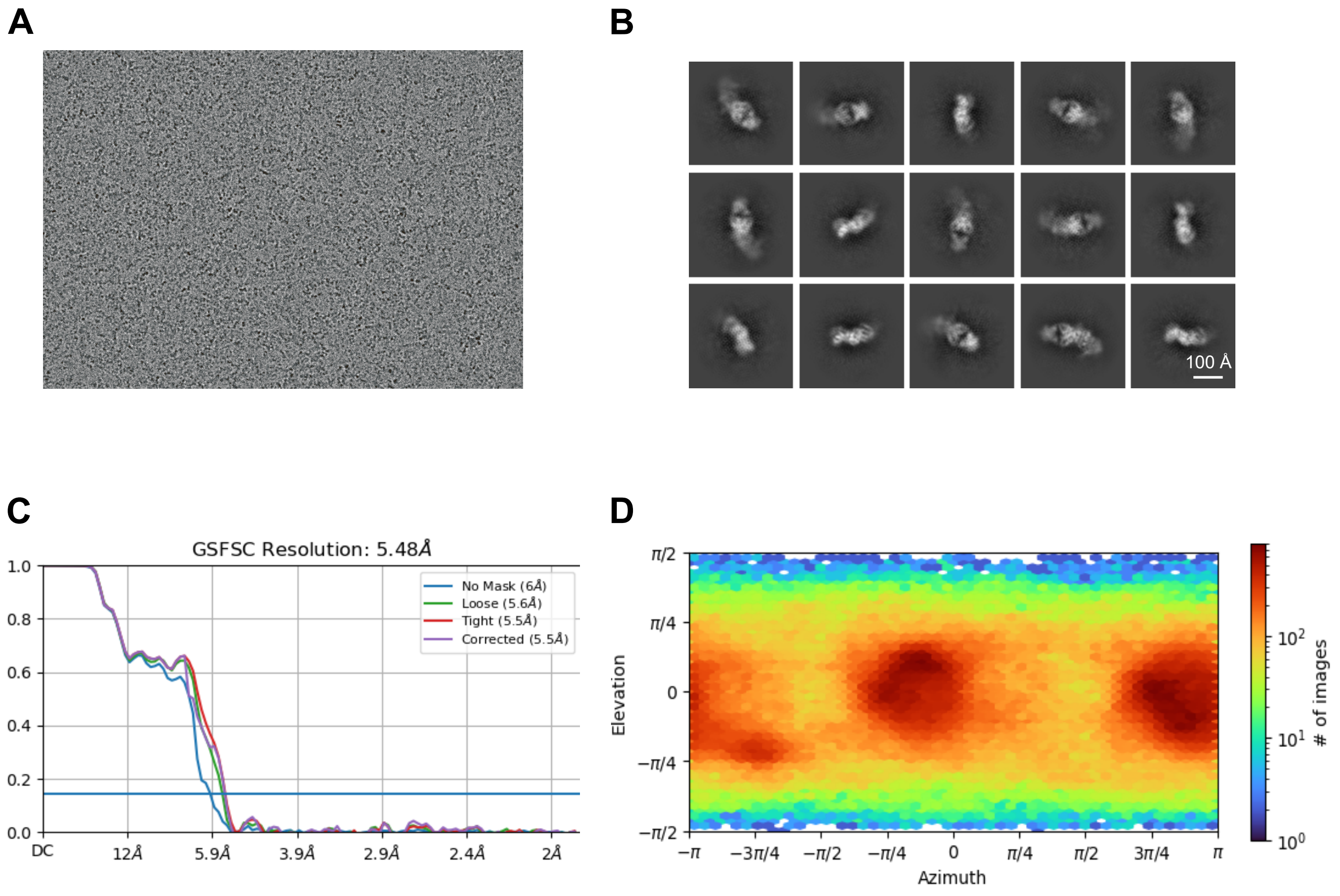


**Figure S3. Cryo-EM details of HIT01-K21Q-R218E with AMG145 Fab at 5.5 Å resolution, related to Figure 3**

(**A**) Representative micrograph.
(**B**) Representative 2D class averages.
(**C**) The gold-standard Fourier shell correlation is shown with the resolution for the density map.
(**D**) The orientations of all particles used in the final refinement are shown as heatmap.

```
Shark fish      --------------------MHLVLVLCLAALAACDYSEDKEVKAPQL--D------HPD         32
Mouse           MGTHCSAWLRWPLLPLLPPLLLLLLLLCPTGAG-A-QDEDGDYEELMLALPSQEDGLADE         58
Cynomolgus      MGTVSSRRSWWPLP---LPL-LLLLLLGPAGARAQ-EDEDGDYEELVLALRSEEDGLADA         55
Human           MGTVSSRRSWWPLP---LLL-LLLLLLGPAGARAQ-EDEDGDYEELVLALRSEEDGLAEA         55
                                      *:*:*  :.      .** : :   *

Shark fish      PGTERVAELLRCTKSVWRIPEQYLVVLREGTRDSHVQRTVSTLRAQAARRGHAIHIMHTY         92
Mouse           AAHVATATFRRCSKEAWRLPGTYIVVLMEETQRLQIEQTAHRLQTRAARRGYVIKVLHIF         118
Cynomolgus      PEHGATATFHRCAKDPWRLPGTYVVVLKEETHRSQSERTARRLQAQAARRGYLTKILHVF         115
Human           PEHGTTATFHRCAKDPWRLPGTYVVVLKEETHLSQSERTARRLQAQAARRGYLTKILHVF         115
                     .* : **:*. **:*  *:*** * *:  : ::*.  *:::*****:  :::* :

Shark fish      SGVFHGFLIKMSSEVLPMALKLPHVAYIEEDSSIFAQGIPWNLERIIQTKHETGKYTPPN         152
Mouse           YDLFPGFLVKMSSDLLGLALKLPHVEYIEEDSFVFAQSIPWNLERIIPAWHQTEEDRSPD         178
Cynomolgus      HHLLPGFLVKMSGDLLELALKLPHVDYIEEDSSVFAQSIPWNLERITPARYRADEYQPPK         175
Human           HGLLPGFLVKMSGDLLELALKLPHVDYIEEDSSVFAQSIPWNLERITPPRYRADEYQPPD         175
                  :: ***:***.::* :******* ****** :***.********    :.: :   *.

Shark fish      DGAQVTVFLLDTSVQTNHREIEGKVMVTDFNSMPEEDGVRVHRQASQCDSHGTHIAGVLS         212
Mouse           GSSQVEVYLLDTSIQGAHREIEGRVTITDFNSVPEEDGTRFHRQASKCDSHGTHLAGVVS         238
Cynomolgus      GGSLVEVYLLDTSIQSDHREIEGRVMVTDFESVPEEDGTRFHRQASKCDSHGTHLAGVVS         235
Human           GGSLVEVYLLDTSIQSDHREIEGRVMVTDFENVPEEDGTRFHRQASKCDSHGTHLAGVVS         235
                ..: * *:*****:*  ******:* :***:.:*****.*.*****:*******:***:*

Shark fish      GRDSGVARGVSVNTVRVLNCQGRGTVSGALAGLEYIRASLQAQPVSPVIILLPFVGGFSR         272
Mouse           GRDAGVAKGTSLHSLRVLNCQGKGTVSGTLIGLEFIRKSQLIQPSGPLVVLLPLAGGYSR         298
Cynomolgus      GRDAGVAKGAGLRSLRVLNCQGKGTVSGTLIGLEFIRKSQLVQPVGPLVVLLPLAGGYSR         295
Human           GRDAGVAKGASMRSLRVLNCQGKGTVSGTLIGLEFIRKSQLVQPVGPLVVLLPLAGGYSR         295
                ***:***:*..:.::*******:*****:* ***:** *   ** .*:::***:.**:**

Shark fish      TLNTACREMVHSGAVLIAAAGNYQDDACMYSPASEPEVITVGASNAADRPLSSGTTGTNL         332
Mouse           ILNAACRHLARTGVVLVAAAGNFRDDACLYSPASAPEVITVGATNAQDQPVTLGTLGTNF         358
Cynomolgus      VFNAACQRLARAGVVLVTAAGNFRDDACLYSPASAPEVITVGATNAQDQPVTLGTLGTNF         355
Human           VLNAACQRLARAGVVLVTAAGNFRDDACLYSPASAPEVITVGATNAQDQPVTLGTLGTNF         355
                 :*:**:.:.::*.**::****::****:***** ********:** *:*:: ** ***:

Shark fish      GRCVDVFAPGDDIISASSDCPTCFTTMSGTSQAAAHVAGIAAVLLNAYPSASPAEVLQLL         392
Mouse           GRCVDLFAPGKDIIGASSDCSTCFMSQSGTSQAAAHVAGIVARMLSREPTLTLAELRQRL         418
Cynomolgus      GRCVDLFAPGEDIIGASSDCSTCFVSRSGTSQAAAHVAGIAAMMLSAEPELTLAELRQRL         415
Human           GRCVDLFAPGEDIIGASSDCSTCFVSQSGTSQAAAHVAGIAAMMLSAEPELTLAELRQRL         415
                *****:****.***.***** *** : ************.* :*.  *  : **: * *

Shark fish      RYHAVQRVINPDSLPPEHYLTTPDMVAALPTS-AATGEKLLCRSVWSKRSGVGSFDTAVA         451
Mouse           IHFSTKDVINMAWFPEDQQVLTPNLVATLPPSTHETGGQLLCRTVWSAHSGPTRTATATA         478
Cynomolgus      IHFSAKDVINEAWFPEDQRVLTPNLVAALPPSTHRAGWQLFCRTVWSAHSGPTRMATAVA         475
Human           IHFSAKDVINEAWFPEDQRVLTPNLVAALPPSTHGAGWQLFCRTVWSAHSGPTRMATAIA         475
                 :.:.: ***   :* :: : **::**:** *   :* :*:**:*** :**     ** *

Shark fish      RCRHGEEMFSCSSYSPNGVHAGERIEIRDGQKVCEAHHGIGGQGVYAIARCCTGSRVKCH         511
Mouse           RCAPEEELLSCSSFSRSGRRRGDWIEAIGGQQVCKALNAFGGEGVYAVARCCLVPRANCS         538
Cynomolgus      RCAQDEELLSCSSFSRSGKRRGERIEAQGGKRVCRAHNAFGGEGVYAIARCCLLPQVNCS         535
Human           RCAPDEELLSCSSFSRSGKRRGERMEAQGGKLVCRAHNAFGGEGVYAIARCCLLPQANCS         535
                **   **::****:* .* : *: :*  .*: **.* :.:**:****:****   :.:*

Shark fish      ASA----SLHVGIDAECPSQEFQLTGCSSHYIRSQDV--AQPSWPLHSNRKACPAGEGGT         565
Mouse           IHNTPAARAGLETHVHCHQKDHVLTGCSFHWEVEDLSVRRQPALRSRRQPGQCVGHQAAS         598
Cynomolgus      VHTAPPAGASMGTRVHCHQQGHVLTGCSSHWEVEDLGTHKPPVLRPRGQPNQCVGHREAS         595
Human           VHTAPPAEASMGTRVHCHQQGHVLTGCSSHWEVEDLGTHKPPVLRPRGQPNQCVGHREAS         595
                          :   ..* .: . ***** *:  .:      *    : :   * . . .:

Shark fish      SHAFCCHAPNLECHLIEHHQSEFTKQVEVSCEDSWTLTGCNAVSHGSVTHAAYTRGNTCV         625
Mouse           VYASCCHAPGLECKIKEHGISGPSEQVTVACEAGWTLTGCNVLPGASLTLGAYSVDNLCV         658
Cynomolgus      IHASCCHAPGLECKVKEHGIPAPQEQVIVACEDGWTLTGCSALPGTSHVLGAYAVDNTCV         655
Human           IHASCCHAPGLECKVKEHGIPAPQEQVTVACEEGWTLTGCSALPGTSHVLGAYAVDNTCV         655
                 :* *****.***:: **      :** *:** .******..:   * . .**: .* **

Shark fish      IQMFGG-------DKGAAAIAICCRYRPLDQQSNNNHEQNT           659
Mouse           ARVHDTARADRTSGEATVAAAICCRSRPSAKASWVQ-----           694
Cynomolgus      VRSQDVSTTGSTSEEAVAAVAICCRSRHLVQASQELQ----           692
Human           VRSRDVSTTGSTSEEAVTAVAICCRSRHLAQASQELQ----           692
                 :  .         :...* ***** *   : *
```

**Figure S4. Amino acid sequence alignment of PCSK9 from difference species, related to Figure 1**

LDLR-contacting residues on human PCSK9 are highlighted in yellow. Common and Latin names with GenBank IDs are as follows: shark fish (*Pangasianodon hypophthalmus*, GenBank # XP_026800391.3); mouse (*Mus musculus*, GenBank # NP_705793.1); Cynomolgous (*Macaca fascicularis*, GenBank # XP_005543314.2 ); human (*Homo sapiens*, GenBank # ABV59216.1).

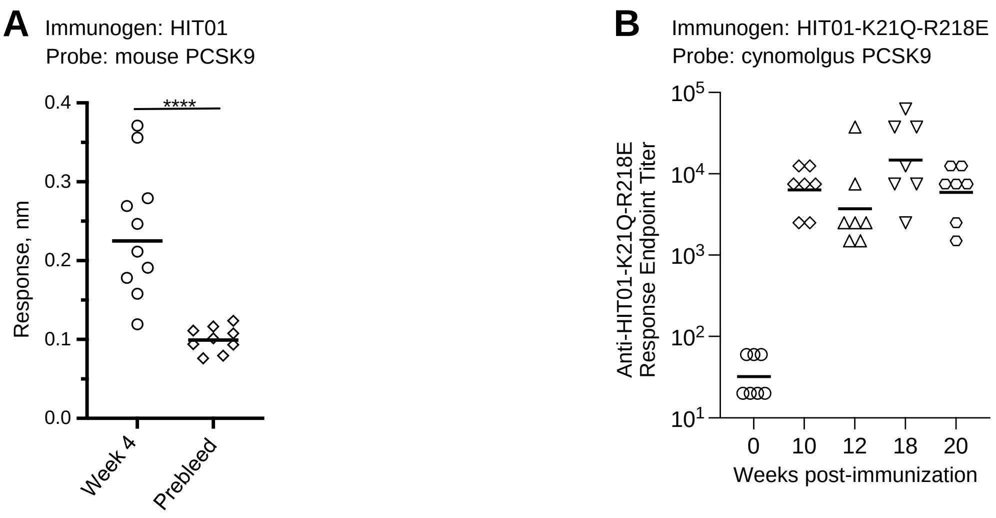


**Figure S5. Serum antibody binding activity to mouse and cynomolgus PCSK9, related to Figures 2 and 4**

(**A**) Mouse serum antibody BLI binding response against mouse PCSK9. *: P<0.05; *: P<0.01; ***: P<0.001; ****: P<0.0001 (Mann Whitney test).

(**B**) Serum antibody ELISA response against cynomolgus PCSK9.

## Table S1. Antigenic assessment of PCSK9 mimics by using ELISA*, related to Figure 1

| PCSK9 mimic constructs# | AMG145 | J16 | Fab33 | Alirocumab | 1D05 |
|---|---|---|---|---|---|
| Cyprinodon_PCSK9_epitope_2_no_identical_9mers_from153_to452_noglycans | 0.055 | 0.096 | 0.042 | 0.047 | 0.059 |
| Pangasianodon_PCSK9_epitope_2_no_identical_9mers_from153_to452_noglycans | 0.055 | 0.089 | 0.038 | 0.048 | 0.057 |
| Anabastestudineus_PCSK9_epitope_2_no_identical_9mers_from153_to452_noglycans | 0.048 | 0.090 | 0.043 | 0.047 | 0.053 |
| Ictaluruspunctatus_PCSK9_epitope_2_no_identical_9mers_from153_to452_noglycans | 0.045 | 0.089 | 0.043 | 0.048 | 0.056 |
| Nototheniacoriiceps_PCSK9_epitope_2_no_identical_9mers_from153_to452_noglycans | 0.046 | 0.090 | 0.044 | 0.048 | 0.056 |
| Pangasianodon_PCSK9_epitope_2_no_identical_9mers_from153_to452_noglycans_rTTHc | 0.046 | 0.088 | 0.042 | 0.084 | 0.051 |
| Ictaluruspunctatus_PCSK9_epitope_2_no_identical_9mers_from153_to452_noglycans_rTTHc | 0.049 | 0.092 | 0.043 | 0.048 | 0.054 |
| Pangasianodon_PCSK9_epitope_1_no_identical_9mers_from153_to452_noglycans_rTTHc | 0.053 | 0.090 | 0.045 | 0.072 | 0.067 |
| Ictaluruspunctatus_PCSK9_epitope_1_no_identical_9mers_from153_to452_noglycans_rTTHc | 0.048 | 0.098 | 0.043 | 0.064 | 0.055 |
| 2b6n_PCSK9_epitope_1_no_identical_9mers_noglycans | 0.047 | 0.092 | 0.043 | 0.049 | 0.056 |
| 2b6n_PCSK9_epitope_1_noglycans | 0.049 | 0.094 | 0.043 | 0.051 | 0.064 |
| 1s2n_PCSK9_epitope_1_no_identical_9mers_noglycans | 0.083 | 0.099 | 0.045 | 0.052 | 0.064 |
| 1s2n_PCSK9_epitope_1_noglycans | 0.051 | 0.093 | 0.047 | 0.048 | 0.057 |
| 3f7o_PCSK9_epitope_1_no_identical_9mers_noglycans | 0.046 | 0.088 | 0.044 | 0.046 | 0.055 |
| 3f7o_PCSK9_epitope_1_noglycans | 0.048 | 0.087 | 0.045 | 0.049 | 0.057 |
| 4dzt_PCSK9_epitope_1_no_identical_9mers_noglycans | 0.047 | 0.086 | 0.034 | 0.055 | 0.062 |
| 4dzt_PCSK9_epitope_1_noglycans | 0.038 | 0.087 | 0.042 | 0.048 | 0.055 |
| 5wsl_PCSK9_epitope_1_no_identical_9mers_noglycans | 0.046 | 0.086 | 0.045 | 0.045 | 0.054 |
| 5wsl_PCSK9_epitope_1_noglycans | 0.045 | 0.090 | 0.043 | 0.045 | 0.054 |
| Cyprinodon_PCSK9_epitope_1_from153_to452_noglycans | 0.047 | 0.083 | 0.043 | 0.049 | 0.059 |
| Cyprinodon_PCSK9_epitope_1_no_identical_9mers_from153_to452_noglycans | 0.050 | 0.088 | 0.045 | 0.046 | 0.056 |
| Anabastestudineus_PCSK9_epitope_1_from153_to452_noglycans | 0.052 | 0.102 | 0.048 | 0.046 | 0.053 |
| Anabastestudineus_PCSK9_epitope_1_no_identical_9mers_from153_to452_noglycans | 0.049 | 0.108 | 0.041 | 0.049 | 0.058 |
| Ictaluruspunctatus_PCSK9_epitope_1_from153_to452_noglycans | 0.059 | 0.093 | 0.044 | 0.053 | 0.062 |
| Ictaluruspunctatus_PCSK9_epitope_1_no_identical_9mers_from153_to452_noglycans | 0.050 | 0.089 | 0.049 | 0.055 | 0.058 |
| Nototheniacoriiceps_PCSK9_epitope_1_from153_to452_noglycans | 0.038 | 0.089 | 0.045 | 0.045 | 0.056 |
| Nototheniacoriiceps_PCSK9_epitope_1_no_identical_9mers_from153_to452_noglycans | 0.062 | 0.094 | 0.042 | 0.045 | 0.056 |
| 2b6n_PCSK9_epitope_1 | 0.046 | 0.089 | 0.034 | 0.047 | 0.063 |
| 2b6n_PCSK9_epitope_1_no_identical_9mers | 0.038 | 0.097 | 0.045 | 0.054 | 0.058 |
| 2b6n_PCSK9_epitope_1_no_identical_9mers_nocat | 0.045 | 0.091 | 0.054 | 0.046 | 0.055 |
| Cyprinodon_PCSK9_epitope_1 | + | + | + | + | + |
| Cyprinodon_PCSK9_epitope_1_no_identical_9mers | 0.048 | 0.091 | 0.044 | 0.048 | 0.059 |
| Cyprinodon_PCSK9_epitope_1_no_identical_9mers_from153 | 0.048 | 0.097 | 0.044 | 0.049 | 0.055 |
| Cyprinodon_PCSK9_epitope_1_no_identical_9mers_from153_to452 | 0.048 | 0.091 | 0.048 | 0.047 | 0.056 |
| Cyprinodon_PCSK9_epitope_1_no_identical_9mers_nocat | 0.045 | 0.090 | 0.044 | 0.049 | 0.056 |
| Cyprinodon_PCSK9_epitope_1_no_identical_9mers_nocat_from153 | 0.048 | 0.094 | 0.048 | 0.051 | 0.068 |
| Cyprinodon_PCSK9_epitope_1_no_identical_9mers_nocat_from153_to452 | 0.049 | 0.101 | 0.042 | 0.077 | 0.070 |
| Pangasianodon_PCSK9_epitope_1 | + | + | + | + | + |
| Pangasianodon_PCSK9_epitope_1_no_identical_9mers (HIT01) | + | + | + | + | + |
| Pangasianodon_PCSK9_epitope_1_no_identical_9mers_from153 | 0.045 | 0.096 | 0.045 | 0.051 | 0.060 |
| Pangasianodon_PCSK9_epitope_1_no_identical_9mers_from153_to452 | 0.046 | 0.095 | 0.045 | 0.062 | 0.057 |
| Pangasianodon_PCSK9_epitope_1_no_identical_9mers_nocat | + | + | 0.099 | 0.052 | + |
| Pangasianodon_PCSK9_epitope_1_no_identical_9mers_nocat_from153 | 0.046 | 0.088 | 0.043 | 0.088 | 0.059 |
| Pangasianodon_PCSK9_epitope_1_no_identical_9mers_nocat_from153_to452 | 0.061 | 0.112 | 0.092 | 0.052 | 0.057 |
| Mus musculus PCSK9 | 0.080 | + | + | 0.124 | + |
| Macaca fascicularis PCSK9 | + | + | + | + | + |
| PCSK9 WT | + | + | + | + | + |
| PCSK9 WT | + | + | + | + | + |
| Medium | 0.048 | 0.093 | 0.046 | 0.064 | 0.080 |

* OD450 values greater than 0.150 were considered positive and are shown as (+).
# 2b6n, 1s2n, 3f7o, 4dzt, 5wsl are PBD codes for protein scaffolds used in each PCSK9 mimics.

## Table S2. Cryo-EM data collection and validation statistics for HIT01-K21Q-R218E with AMG145 Fab, related to Figure 3

| | **HIT01-K21Q-R218E with AMG145 Fab** (EMD-41409) |
|---|---|
| **Data collection and processing** | |
| Magnification | 45,000 |
| Voltage (kV) | 200 |
| Electron exposure ($e^{-}$/Å$^2$) | 66.47 |
| Defocus range (μm) | -1.0 to -2.5 |
| Pixel size (Å) | 0.92 |
| Symmetry imposed | C1 |
| Final particle images (no.) | 328K |
| Map resolution (Å) | 5.48 |
| FSC threshold | 0.143 |

**Table S3. NHP information, related to Figure 4**

| Animal ID | Gender | Date of birth | Age at week 3 (1/14/2021) |
|---|---|---|---|
| NHP-1 | M | 12/28/2014 | 7 |
| NHP-2 | F | 8/10/2015 | 6 |
| NHP-3 | M | 7/01/2014 | 7 |
| NHP-4 | M | 3/04/2014 | 7 |
| NHP-5 | M | 6/20/2015 | 6 |
| NHP-6 | F | 11/28/2014 | 7 |
| NHP-7 | F | 11/1/2014 | 7 |

# Table S4. NHP cholesterol measurement data, related to Figure 4

Cholesterol concentrations are in mg/dL. Day 1 is defined as the day when the first immunization occurred.

NHP-1

| Day | CHOL | HDL | LDL |
|---|---|---|---|
| -190 | 141 | 44 | 100 |
| -190 | 140 | 45 | 99 |
| -190 | 144 | 47 | 100 |
| -176 | 134 | 46 | 87 |
| -176 | 132 | 44 | 87 |
| -162 | 130 | 56 | 67 |
| -162 | 131 | 55 | 67 |
| -162 | 129 | 58 | 65 |
| -84 | 132 | 44 | 80 |
| -84 | 129 | 45 | 78 |
| -84 | 126 | 46 | 81 |
| -77 | 130 | 45 | 78 |
| -77 | 129 | 44 | 77 |
| -77 | 129 | 47 | 77 |
| -40 | 116 | 43 | 74 |
| -40 | 130 | 44 | 77 |
| -40 | 131 | 45 | 77 |
| -40 | 105 | 38 | 63 |
| -25 | 126 | 45 | 72 |
| -25 | 127 | 43 | 72 |
| -25 | 126 | 44 | 74 |
| -25 | 124 | 45 | 71 |
| 1 | 149 | 48 | 104 |
| 1 | 145 | 47 | 98 |
| 1 | 153 | 47 | 102 |
| 15 | 129 | 42 | 83 |
| 15 | 140 | 43 | 88 |
| 15 | 118 | 37 | 73 |
| 15 | 137 | 44 | 85 |
| 29 | 145 | 46 | 100 |
| 29 | 176 | 53 | 116 |
| 29 | 134 | 40 | 88 |
| 43 | 124 | 46 | 80 |
| 43 | 145 | 51 | 93 |
| 43 | 125 | 43 | 77 |
| 43 | 127 | 45 | 80 |
| 71 | 163 | 56 | 104 |
| 71 | 161 | 55 | 100 |
| 71 | 156 | 55 | 99 |
| 85 | 150 | 50 | 95 |
| 85 | 150 | 52 | 96 |
| 85 | 147 | 50 | 92 |
| 99 | 136 | 47 | 81 |
| 99 | 137 | 47 | 81 |
| 99 | 133 | 48 | 80 |
| 127 | 154 | 55 | 94 |
| 127 | 157 | 56 | 95 |
| 127 | 151 | 56 | 93 |
| 141 | 142 | 51 | 81 |
| 141 | 142 | 51 | 79 |
| 141 | 138 | 51 | 79 |
| 155 | 135 | 48 | 77 |
| 155 | 136 | 48 | 76 |
| 155 | 131 | 48 | 76 |
| 169 | 140 | 48 | 83 |
| 169 | 143 | 47 | 84 |
| 169 | 135 | 49 | 83 |
| 183 | 140 | 52 | 84 |
| 183 | 140 | 53 | 82 |
| 183 | 139 | 53 | 82 |
| 197 | 148 | 47 | 99 |
| 197 | 150 | 48 | 98 |
| 197 | 144 | 48 | 98 |
| 211 | 163 | 53 | 107 |
| 211 | 156 | 53 | 106 |
| 211 | 154 | 53 | 109 |
| 225 | 163 | 50 | 111 |
| 225 | 160 | 48 | 116 |
| 225 | 163 | 48 | 115 |
| 239 | 154 | 51 | 101 |
| 239 | 153 | 49 | 101 |
| 239 | 155 | 50 | 104 |
| 253 | 143 | 45 | 96 |
| 253 | 142 | 45 | 96 |
| 253 | 146 | 44 | 96 |
| 281 | 151 | 48 | 99 |
| 281 | 149 | 48 | 101 |
| 281 | 150 | 49 | 99 |
| 295 | 162 | 52 | 110 |
| 295 | 162 | 53 | 111 |
| 295 | 167 | 55 | 109 |
| 309 | 157 | 46 | 104 |
| 309 | 158 | 46 | 104 |
| 309 | 160 | 49 | 104 |
| 323 | 163 | 49 | 119 |
| 323 | 163 | 49 | 121 |
| 323 | 167 | 52 | 119 |
| 337 | 163 | 50 | 117 |
| 337 | 161 | 49 | 116 |
| 337 | 167 | 54 | 117 |
| 351 | 144 | 47 | 95 |
| 351 | 144 | 48 | 95 |
| 351 | 147 | 51 | 96 |
| 365 | 169 | 50 | 131 |
| 365 | 169 | 50 | 131 |
| 365 | 171 | 54 | 134 |

NHP-2

| Day | CHOL | HDL | LDL |
|---|---|---|---|
| -190 | 165 | 77 | 85 |
| -190 | 161 | 77 | 83 |
| -190 | 163 | 79 | 82 |
| -176 | 126 | 62 | 58 |
| -176 | 126 | 60 | 59 |
| -162 | 141 | 76 | 54 |
| -162 | 136 | 72 | 51 |
| -162 | 141 | 77 | 53 |
| -84 | 142 | 69 | 64 |
| -84 | 139 | 69 | 62 |
| -40 | 123 | 62 | 61 |
| -40 | 122 | 58 | 61 |
| -40 | 121 | 57 | 58 |
| -40 | 122 | 60 | 58 |
| -25 | 116 | 60 | 47 |
| -25 | 118 | 59 | 48 |
| -25 | 121 | 62 | 50 |
| -25 | 102 | 51 | 42 |
| 1 | 137 | 79 | 56 |
| 1 | 157 | 85 | 62 |
| 1 | 154 | 82 | 61 |
| 1 | 126 | 67 | 48 |
| 15 | 128 | 71 | 54 |
| 15 | 142 | 73 | 56 |
| 15 | 122 | 62 | 49 |
| 15 | 128 | 69 | 52 |
| 29 | 113 | 68 | 42 |
| 29 | 128 | 69 | 46 |
| 29 | 116 | 64 | 43 |
| 29 | 115 | 63 | 41 |
| 43 | 121 | 70 | 47 |
| 43 | 144 | 77 | 56 |
| 43 | 114 | 62 | 45 |
| 43 | 130 | 71 | 50 |
| 71 | 131 | 64 | 62 |
| 71 | 130 | 63 | 59 |
| 71 | 128 | 64 | 60 |
| 85 | 155 | 86 | 55 |
| 85 | 156 | 86 | 56 |
| 85 | 151 | 85 | 55 |
| 99 | 130 | 73 | 48 |
| 99 | 127 | 71 | 45 |
| 99 | 121 | 69 | 43 |
| 127 | 116 | 59 | 42 |
| 127 | 118 | 60 | 43 |
| 127 | 113 | 60 | 42 |
| 141 | 152 | 73 | 70 |
| 141 | 155 | 75 | 71 |
| 141 | 152 | 73 | 69 |
| 155 | 113 | 68 | 31 |
| 155 | 114 | 70 | 30 |
| 155 | 110 | 70 | 31 |
| 169 | 120 | 68 | 41 |
| 169 | 123 | 70 | 41 |
| 169 | 118 | 67 | 41 |
| 183 | 107 | 63 | 33 |
| 183 | 106 | 64 | 32 |
| 183 | 105 | 64 | 32 |
| 197 | 134 | 71 | 52 |
| 197 | 135 | 72 | 52 |
| 197 | 130 | 73 | 51 |
| 211 | 120 | 66 | 42 |
| 211 | 117 | 68 | 43 |
| 211 | 115 | 64 | 43 |
| 225 | 144 | 82 | 52 |
| 225 | 141 | 80 | 54 |
| 225 | 144 | 78 | 54 |
| 239 | 124 | 75 | 42 |
| 239 | 122 | 71 | 44 |
| 239 | 123 | 72 | 44 |
| 253 | 128 | 73 | 49 |
| 253 | 129 | 74 | 48 |
| 253 | 129 | 74 | 49 |
| 281 | 121 | 68 | 43 |
| 281 | 120 | 69 | 44 |
| 281 | 122 | 73 | 44 |
| 295 | 122 | 64 | 55 |
| 295 | 122 | 64 | 55 |
| 295 | 126 | 67 | 54 |
| 309 | 130 | 67 | 60 |
| 309 | 129 | 67 | 60 |
| 309 | 133 | 72 | 60 |
| 323 | 129 | 67 | 57 |
| 323 | 129 | 67 | 57 |
| 323 | 136 | 72 | 57 |
| 337 | 122 | 64 | 55 |
| 337 | 121 | 63 | 55 |
| 337 | 126 | 70 | 56 |
| 351 | 116 | 63 | 51 |
| 351 | 117 | 62 | 51 |
| 351 | 122 | 68 | 50 |
| 365 | 124 | 67 | 52 |
| 365 | 125 | 66 | 52 |
| 365 | 126 | 70 | 51 |

NHP-3

| Day | CHOL | HDL | LDL |
|---|---|---|---|
| -190 | 134 | 63 | 62 |
| -190 | 133 | 63 | 61 |
| -190 | 136 | 66 | 63 |
| -176 | 104 | 52 | 45 |
| -162 | 109 | 55 | 46 |
| -162 | 108 | 54 | 44 |
| -162 | 111 | 57 | 45 |
| -84 | 131 | 65 | 54 |
| -84 | 117 | 59 | 48 |
| -40 | 105 | 58 | 42 |
| -40 | 107 | 55 | 40 |
| -40 | 105 | 54 | 39 |
| -25 | 118 | 60 | 45 |
| -25 | 126 | 62 | 48 |
| -25 | 113 | 55 | 43 |
| -25 | 119 | 59 | 44 |
| 1 | 128 | 65 | 57 |
| 1 | 131 | 63 | 58 |
| 1 | 128 | 65 | 57 |
| 1 | 129 | 64 | 56 |
| 15 | 118 | 60 | 54 |
| 15 | 125 | 61 | 57 |
| 15 | 113 | 54 | 50 |
| 15 | 122 | 62 | 53 |
| 29 | 118 | 63 | 49 |
| 29 | 142 | 70 | 58 |
| 29 | 114 | 53 | 47 |
| 29 | 122 | 62 | 50 |
| 43 | 100 | 56 | 38 |
| 43 | 110 | 59 | 44 |
| 43 | 104 | 55 | 40 |
| 43 | 97 | 52 | 37 |
| 71 | 112 | 58 | 45 |
| 71 | 109 | 58 | 43 |
| 71 | 109 | 59 | 45 |
| 85 | 128 | 65 | 52 |
| 85 | 130 | 65 | 51 |
| 85 | 127 | 64 | 51 |
| 99 | 111 | 54 | 45 |
| 99 | 110 | 53 | 44 |
| 99 | 105 | 53 | 43 |
| 127 | 120 | 59 | 51 |
| 127 | 121 | 61 | 52 |
| 127 | 118 | 61 | 51 |
| 141 | 120 | 61 | 48 |
| 141 | 63 | 39 | 17 |
| 141 | 117 | 61 | 47 |
| 155 | 106 | 52 | 46 |
| 155 | 108 | 53 | 46 |
| 155 | 103 | 53 | 45 |
| 169 | 114 | 57 | 50 |
| 169 | 117 | 58 | 50 |
| 169 | 111 | 58 | 49 |
| 183 | 113 | 56 | 50 |
| 183 | 113 | 55 | 49 |
| 183 | 112 | 56 | 48 |
| 197 | 123 | 60 | 57 |
| 197 | 127 | 61 | 57 |
| 197 | 123 | 60 | 55 |
| 211 | 119 | 58 | 53 |
| 211 | 116 | 57 | 53 |
| 211 | 114 | 56 | 55 |
| 225 | 132 | 68 | 59 |
| 225 | 132 | 65 | 60 |
| 225 | 131 | 65 | 61 |
| 239 | 125 | 64 | 52 |
| 239 | 124 | 62 | 52 |
| 239 | 124 | 62 | 52 |
| 253 | 117 | 62 | 51 |
| 253 | 117 | 61 | 50 |
| 253 | 119 | 61 | 51 |
| 281 | 126 | 62 | 57 |
| 281 | 127 | 62 | 56 |
| 281 | 129 | 66 | 55 |
| 295 | 130 | 64 | 60 |
| 295 | 127 | 64 | 60 |
| 295 | 134 | 68 | 59 |
| 309 | 114 | 59 | 46 |
| 309 | 115 | 60 | 46 |
| 309 | 117 | 64 | 46 |
| 323 | 120 | 58 | 56 |
| 323 | 117 | 58 | 57 |
| 323 | 121 | 63 | 57 |
| 337 | 131 | 63 | 64 |
| 337 | 131 | 62 | 64 |
| 337 | 139 | 70 | 66 |
| 351 | 124 | 60 | 56 |
| 351 | 124 | 59 | 57 |
| 351 | 126 | 65 | 58 |
| 365 | 122 | 58 | 59 |
| 365 | 123 | 59 | 59 |
| 365 | 126 | 63 | 58 |

NHP-4

| Day | CHOL | HDL | LDL |
|---|---|---|---|
| -190 | 134 | 53 | 79 |
| -190 | 127 | 51 | 76 |
| -190 | 131 | 56 | 76 |
| -176 | 119 | 53 | 60 |
| -176 | 115 | 53 | 59 |
| -162 | 126 | 51 | 72 |
| -162 | 125 | 51 | 71 |
| -162 | 126 | 53 | 70 |
| -84 | 134 | 56 | 72 |
| -84 | 124 | 52 | 67 |
| -77 | 131 | 58 | 68 |
| -77 | 124 | 52 | 63 |
| -40 | 136 | 62 | 71 |
| -40 | 136 | 59 | 69 |
| -40 | 136 | 60 | 69 |
| -40 | 129 | 56 | 65 |
| -25 | 133 | 62 | 68 |
| -25 | 131 | 58 | 64 |
| -25 | 139 | 65 | 68 |
| -25 | 120 | 55 | 59 |
| 1 | 148 | 65 | 87 |
| 1 | 142 | 62 | 80 |
| 1 | 146 | 60 | 82 |
| 15 | 114 | 57 | 54 |
| 15 | 124 | 59 | 56 |
| 15 | 103 | 48 | 47 |
| 15 | 118 | 59 | 53 |
| 29 | 133 | 60 | 72 |
| 29 | 154 | 66 | 82 |
| 29 | 128 | 53 | 69 |
| 29 | 134 | 58 | 70 |
| 43 | 93 | 54 | 35 |
| 43 | 105 | 59 | 38 |
| 43 | 96 | 52 | 35 |
| 43 | 93 | 52 | 34 |
| 71 | 128 | 61 | 60 |
| 71 | 128 | 62 | 59 |
| 71 | 125 | 61 | 58 |
| 85 | 135 | 64 | 64 |
| 85 | 136 | 65 | 63 |
| 85 | 131 | 64 | 65 |
| 99 | 89 | 52 | 27 |
| 99 | 89 | 53 | 27 |
| 99 | 86 | 52 | 26 |
| 127 | 124 | 59 | 57 |
| 127 | 126 | 59 | 56 |
| 127 | 121 | 60 | 57 |
| 141 | 127 | 62 | 57 |
| 141 | 85 | 49 | 29 |
| 141 | 125 | 63 | 55 |
| 155 | 83 | 54 | 20 |
| 155 | 83 | 54 | 20 |
| 155 | 80 | 54 | 19 |
| 169 | 107 | 61 | 38 |
| 169 | 109 | 62 | 37 |
| 169 | 102 | 62 | 37 |
| 183 | 114 | 57 | 41 |
| 183 | 114 | 59 | 41 |
| 183 | 110 | 58 | 41 |
| 197 | 131 | 64 | 63 |
| 197 | 134 | 65 | 63 |
| 197 | 129 | 63 | 63 |
| 211 | 132 | 64 | 59 |
| 211 | 129 | 66 | 59 |
| 211 | 126 | 64 | 62 |
| 225 | 143 | 62 | 74 |
| 225 | 143 | 62 | 75 |
| 225 | 142 | 61 | 75 |
| 239 | 132 | 63 | 66 |
| 239 | 131 | 60 | 65 |
| 239 | 133 | 61 | 66 |
| 253 | 127 | 61 | 63 |
| 253 | 126 | 61 | 62 |
| 253 | 128 | 62 | 63 |
| 281 | 136 | 64 | 66 |
| 281 | 132 | 64 | 66 |
| 281 | 133 | 67 | 66 |
| 295 | 125 | 63 | 60 |
| 295 | 126 | 62 | 60 |
| 295 | 130 | 67 | 59 |
| 309 | 124 | 59 | 64 |
| 309 | 124 | 58 | 63 |
| 309 | 127 | 62 | 64 |
| 323 | 125 | 57 | 66 |
| 323 | 125 | 58 | 66 |
| 323 | 129 | 62 | 66 |
| 337 | 125 | 57 | 68 |
| 337 | 123 | 58 | 68 |
| 337 | 129 | 63 | 68 |
| 351 | 122 | 55 | 67 |
| 351 | 120 | 54 | 66 |
| 351 | 126 | 59 | 66 |
| 365 | 130 | 56 | 73 |
| 365 | 129 | 57 | 72 |
| 365 | 132 | 59 | 71 |

NHP-5

| Day | CHOL | HDL | LDL |
|---|---|---|---|
| -190 | 109 | 57 | 47 |
| -190 | 106 | 56 | 46 |
| -190 | 109 | 60 | 47 |
| -176 | 72 | 40 | 26 |
| -176 | 84 | 48 | 31 |
| -162 | 87 | 48 | 36 |
| -162 | 87 | 47 | 35 |
| -162 | 92 | 52 | 37 |
| -84 | 91 | 50 | 35 |
| -84 | 88 | 49 | 34 |
| -84 | 88 | 51 | 34 |
| -77 | 87 | 47 | 35 |
| -77 | 88 | 46 | 35 |
| -40 | 84 | 48 | 31 |
| -40 | 88 | 49 | 31 |
| -40 | 78 | 43 | 27 |
| -40 | 80 | 46 | 28 |
| -25 | 87 | 50 | 35 |
| -25 | 86 | 47 | 33 |
| -25 | 86 | 48 | 33 |
| 1 | 96 | 54 | 40 |
| 1 | 106 | 57 | 43 |
| 1 | 91 | 51 | 38 |
| 1 | 91 | 51 | 36 |
| 15 | 81 | 50 | 27 |
| 15 | 93 | 53 | 31 |
| 15 | 73 | 42 | 25 |
| 15 | 80 | 48 | 26 |
| 29 | 87 | 48 | 38 |
| 29 | 92 | 47 | 39 |
| 29 | 103 | 53 | 41 |
| 29 | 83 | 44 | 34 |
| 43 | 67 | 45 | 17 |
| 43 | 74 | 49 | 19 |
| 43 | 64 | 43 | 16 |
| 43 | 67 | 46 | 17 |
| 71 | 85 | 48 | 29 |
| 71 | 88 | 50 | 29 |
| 71 | 79 | 47 | 27 |
| 85 | 84 | 52 | 26 |
| 85 | 86 | 52 | 26 |
| 85 | 83 | 53 | 26 |
| 99 | 68 | 44 | 18 |
| 99 | 69 | 44 | 18 |
| 99 | 67 | 44 | 18 |
| 127 | 83 | 48 | 28 |
| 127 | 84 | 51 | 27 |
| 127 | 82 | 49 | 28 |
| 141 | 84 | 49 | 30 |
| 141 | 129 | 64 | 55 |
| 141 | 81 | 50 | 29 |
| 155 | 73 | 44 | 20 |
| 155 | 73 | 45 | 20 |
| 155 | 71 | 45 | 20 |
| 169 | 88 | 50 | 33 |
| 169 | 88 | 50 | 32 |
| 169 | 85 | 49 | 32 |
| 183 | 90 | 49 | 32 |
| 183 | 90 | 50 | 32 |
| 183 | 89 | 51 | 32 |
| 197 | 101 | 56 | 41 |
| 197 | 104 | 56 | 39 |
| 197 | 102 | 56 | 40 |
| 211 | 105 | 57 | 41 |
| 211 | 102 | 57 | 41 |
| 211 | 100 | 56 | 42 |
| 225 | 100 | 56 | 37 |
| 225 | 98 | 54 | 39 |
| 225 | 100 | 54 | 39 |
| 239 | 86 | 51 | 30 |
| 239 | 86 | 48 | 31 |
| 239 | 87 | 49 | 31 |
| 253 | 83 | 46 | 32 |
| 253 | 84 | 47 | 31 |
| 253 | 85 | 47 | 32 |
| 281 | 95 | 55 | 35 |
| 281 | 94 | 53 | 34 |
| 281 | 96 | 58 | 36 |
| 295 | 96 | 54 | 40 |
| 295 | 97 | 53 | 40 |
| 295 | 99 | 58 | 40 |
| 309 | 87 | 53 | 28 |
| 309 | 86 | 53 | 28 |
| 309 | 88 | 56 | 28 |
| 323 | 88 | 51 | 33 |
| 323 | 88 | 50 | 32 |
| 323 | 91 | 55 | 32 |
| 337 | 91 | 53 | 37 |
| 337 | 92 | 53 | 37 |
| 337 | 96 | 57 | 38 |
| 351 | 79 | 48 | 30 |
| 351 | 80 | 48 | 30 |
| 351 | 88 | 51 | 30 |
| 365 | 78 | 45 | 32 |
| 365 | 80 | 45 | 32 |
| 365 | 80 | 48 | 32 |

NHP-6

| Day | CHOL | HDL | LDL |
|---|---|---|---|
| -190 | 96 | 48 | 46 |
| -190 | 92 | 47 | 44 |
| -190 | 97 | 50 | 45 |
| -176 | 86 | 49 | 31 |
| -162 | 93 | 49 | 36 |
| -162 | 90 | 48 | 35 |
| -162 | 91 | 51 | 35 |
| -84 | 90 | 49 | 33 |
| -84 | 73 | 40 | 26 |
| -84 | 74 | 44 | 28 |
| -77 | 93 | 50 | 36 |
| -77 | 91 | 50 | 34 |
| -77 | 91 | 50 | 34 |
| -40 | 86 | 46 | 35 |
| -40 | 97 | 50 | 39 |
| -40 | 72 | 37 | 29 |
| -40 | 85 | 45 | 34 |
| -25 | 79 | 47 | 23 |
| -25 | 88 | 50 | 25 |
| -25 | 70 | 40 | 20 |
| -25 | 83 | 47 | 24 |
| 1 | 91 | 55 | 36 |
| 1 | 100 | 56 | 37 |
| 1 | 105 | 58 | 36 |
| 1 | 81 | 47 | 30 |
| 15 | 88 | 50 | 31 |
| 15 | 96 | 52 | 33 |
| 15 | 86 | 47 | 29 |
| 15 | 83 | 47 | 29 |
| 29 | 71 | 45 | 24 |
| 29 | 88 | 50 | 28 |
| 29 | 68 | 40 | 21 |
| 29 | 78 | 46 | 25 |
| 43 | 67 | 50 | 12 |
| 43 | 76 | 54 | 13 |
| 43 | 67 | 47 | 11 |
| 43 | 65 | 47 | 11 |
| 71 | 89 | 55 | 28 |
| 71 | 78 | 48 | 23 |
| 71 | 79 | 50 | 25 |
| 85 | 82 | 52 | 19 |
| 85 | 83 | 52 | 18 |
| 85 | 80 | 52 | 18 |
| 99 | 58 | 43 | 8 |
| 99 | 60 | 43 | 8 |
| 99 | 57 | 43 | 7 |
| 127 | 62 | 40 | 15 |
| 127 | 63 | 42 | 14 |
| 127 | 60 | 42 | 14 |
| 141 | 59 | 36 | 16 |
| 141 | 121 | 62 | 48 |
| 141 | 58 | 37 | 16 |
| 155 | 66 | 44 | 15 |
| 155 | 67 | 45 | 14 |
| 155 | 64 | 45 | 14 |
| 169 | 64 | 42 | 14 |
| 169 | 64 | 42 | 14 |
| 169 | 62 | 43 | 13 |
| 183 | 62 | 41 | 13 |
| 183 | 63 | 41 | 13 |
| 183 | 60 | 41 | 13 |
| 197 | 80 | 39 | 24 |
| 197 | 84 | 40 | 24 |
| 197 | 82 | 39 | 23 |
| 211 | 70 | 41 | 22 |
| 211 | 68 | 42 | 22 |
| 211 | 67 | 41 | 22 |
| 225 | 72 | 45 | 22 |
| 225 | 72 | 43 | 22 |
| 225 | 70 | 43 | 22 |
| 239 | 77 | 44 | 23 |
| 239 | 76 | 42 | 23 |
| 239 | 76 | 44 | 23 |
| 253 | 71 | 42 | 20 |
| 253 | 71 | 42 | 20 |
| 253 | 73 | 43 | 20 |
| 281 | 81 | 47 | 28 |
| 281 | 82 | 46 | 27 |
| 281 | 83 | 50 | 27 |
| 295 | 80 | 43 | 29 |
| 295 | 79 | 44 | 29 |
| 295 | 81 | 47 | 29 |
| 309 | 73 | 41 | 21 |
| 309 | 73 | 40 | 21 |
| 309 | 74 | 42 | 21 |
| 323 | 83 | 43 | 34 |
| 323 | 81 | 42 | 34 |
| 323 | 86 | 47 | 34 |
| 337 | 93 | 50 | 42 |
| 337 | 93 | 50 | 42 |
| 337 | 97 | 54 | 42 |
| 351 | 94 | 48 | 32 |
| 351 | 92 | 48 | 32 |
| 351 | 96 | 51 | 31 |
| 365 | 101 | 53 | 41 |
| 365 | 103 | 53 | 40 |
| 365 | 104 | 57 | 41 |

NHP-7

| Day | CHOL | HDL | LDL |
|---|---|---|---|
| -190 | 165 | 60 | 106 |
| -190 | 163 | 60 | 107 |
| -190 | 171 | 62 | 108 |
| -176 | 174 | 65 | 108 |
| -162 | 169 | 82 | 78 |
| -162 | 168 | 81 | 76 |
| -162 | 169 | 85 | 77 |
| -84 | 162 | 67 | 84 |
| -84 | 152 | 62 | 79 |
| -84 | 153 | 65 | 78 |
| -77 | 170 | 76 | 86 |
| -77 | 160 | 71 | 81 |
| -77 | 163 | 73 | 80 |
| -40 | 160 | 63 | 96 |
| -40 | 171 | 65 | 98 |
| -40 | 147 | 55 | 85 |
| -40 | 153 | 59 | 89 |
| -25 | 140 | 60 | 84 |
| -25 | 161 | 66 | 92 |
| -25 | 126 | 50 | 70 |
| -25 | 131 | 54 | 73 |
| 1 | 155 | 62 | 98 |
| 1 | 177 | 67 | 107 |
| 1 | 175 | 66 | 104 |
| 1 | 134 | 52 | 83 |
| 15 | 150 | 80 | 65 |
| 15 | 167 | 81 | 71 |
| 15 | 140 | 69 | 58 |
| 15 | 151 | 72 | 62 |
| 29 | 151 | 71 | 81 |
| 29 | 175 | 74 | 91 |
| 29 | 157 | 67 | 80 |
| 29 | 160 | 69 | 82 |
| 43 | 159 | 78 | 80 |
| 43 | 180 | 81 | 89 |
| 43 | 166 | 74 | 81 |
| 43 | 148 | 67 | 75 |
| 71 | 219 | 79 | 140 |
| 71 | 208 | 75 | 131 |
| 71 | 203 | 74 | 132 |
| 85 | 171 | 63 | 100 |
| 85 | 170 | 64 | 99 |
| 85 | 165 | 63 | 99 |
| 99 | 142 | 70 | 60 |
| 99 | 142 | 70 | 59 |
| 99 | 137 | 69 | 58 |
| 127 | 152 | 69 | 74 |
| 127 | 153 | 73 | 71 |
| 127 | 148 | 69 | 71 |
| 141 | 163 | 67 | 79 |
| 141 | 166 | 67 | 79 |
| 141 | 158 | 67 | 77 |
| 155 | 132 | 63 | 60 |
| 155 | 133 | 63 | 58 |
| 155 | 127 | 64 | 58 |
| 169 | 141 | 64 | 66 |
| 169 | 141 | 64 | 65 |
| 169 | 138 | 65 | 64 |
| 183 | 154 | 65 | 72 |
| 183 | 155 | 66 | 71 |
| 183 | 152 | 64 | 70 |
| 197 | 146 | 64 | 73 |
| 197 | 152 | 65 | 74 |
| 197 | 145 | 64 | 72 |
| 211 | 167 | 61 | 98 |
| 211 | 161 | 62 | 96 |
| 211 | 156 | 59 | 99 |
| 225 | 155 | 62 | 81 |
| 225 | 152 | 59 | 84 |
| 225 | 153 | 59 | 84 |
| 239 | 148 | 69 | 65 |
| 239 | 148 | 65 | 66 |
| 239 | 147 | 66 | 66 |
| 253 | 162 | 56 | 107 |
| 253 | 159 | 57 | 109 |
| 253 | 164 | 56 | 108 |
| 281 | 158 | 75 | 68 |
| 281 | 156 | 73 | 68 |
| 281 | 158 | 79 | 69 |
| 295 | 155 | 71 | 76 |
| 295 | 156 | 71 | 77 |
| 295 | 161 | 76 | 76 |
| 309 | 138 | 56 | 79 |
| 309 | 137 | 56 | 76 |
| 309 | 141 | 61 | 76 |
| 323 | 139 | 65 | 66 |
| 323 | 139 | 64 | 67 |
| 323 | 144 | 71 | 66 |
| 337 | 146 | 56 | 91 |
| 337 | 145 | 55 | 92 |
| 337 | 154 | 61 | 92 |
| 351 | 161 | 69 | 82 |
| 351 | 158 | 70 | 81 |
| 351 | 164 | 74 | 82 |
| 365 | 159 | 69 | 81 |
| 365 | 158 | 69 | 81 |
| 365 | 161 | 74 | 80 |

## Table S5. Longitudinal quantification of serum PCSK9 concentration (raw data), related to Figure 5

| NHP-1 | Series 1 | | | | Series 2 | | | |
|---|---|---|---|---|---|---|---|---|
| PreBleed (08/20) | 301 | 320 | 321 | 443 | 304 | 289 | 317 | 468 |
| PreBleed (08/20) | 145 | 127 | | | 140 | 126 | 227 | |
| PreBleed (09/20) | 271 | 271 | 306 | 299 | 217 | 229 | 223 | 374 |
| PreBleed (12/20) | 182 | 154 | | | 133 | 91 | | |
| PreBleed Wk-3 | 426 | 386 | 273 | | 275 | 243 | 217 | |
| PreBleed WK-1 | 155 | 260 | 316 | | 148 | 218 | 353 | |
| Wk 4 | 491 | 608 | | | 400 | 406 | 521 | |
| Wk 6 | 1643 | 1972 | | | 1613 | 2074 | | |
| Wk 12 | 1215 | 1240 | | | 1184 | 1357 | | |
| Wk 14 | | 1988 | | | 1154 | 1792 | | |
| Wk 18 | 1334 | 1444 | | | 1364 | 1596 | | |
| Wk 20 | 1792 | 2200 | | | 1708 | 2200 | | |
| WK 22 | 2484 | 2060 | | | 797 | 1984 | 2120 | |
| Wk 24 | 2811 | 3334 | | | 2898 | 3719 | | |
| Wk 26 | 2726 | 3581 | | | 2624 | 3939 | | |

| NHP-2 | Series 1 | | | | Series 2 | | | |
|---|---|---|---|---|---|---|---|---|
| PreBleed (08/20) | 179 | 226 | 200 | 245 | 213 | 191 | 209 | 337 |
| PreBleed (08/20) | 116 | | | | 86 | | | |
| PreBleed (9/20) | 161 | 164 | 150 | 158 | 153 | 137 | 179 | 205 |
| PreBleed (9/20) | 125 | 84 | | | 98 | | | |
| PreBleed (12/20) | 118 | 72 | | | 109 | | | |
| PreBleed Wk-3 | 203 | 206 | | | 181 | 117 | | |
| PreBleed WK-1 | 319 | | 178 | | 227 | 238 | | |
| PreBleed Wk0 | 174 | 111 | | | 178 | 93 | 151 | |
| Wk 4 | 400 | 437 | | | 395 | 449 | 461 | |
| Wk 6 | 1116 | 1471 | | | 1198 | 1440 | | |
| Wk 12 | 564 | 685 | 708 | | 512 | 690 | 804 | |
| Wk 14 | 2392 | 2320 | | | 1001 | 992 | | |
| Wk 18 | 674 | 426 | 420 | | 628 | 435 | 376 | |
| Wk 20 | 750 | 1044 | 1160 | | 683 | 996 | 1200 | |
| Wk 22 | 1040 | | | | 435 | 692 | | |
| Wk 24 | 531 | 582 | 564 | | 532 | 602 | 636 | |
| Wk 26 | 629 | 756 | 927 | | 577 | 647 | 1063 | |

| NHP-3 | Series 1 | | | | Series 2 | | | |
|---|---|---|---|---|---|---|---|---|
| PreBleed (08/20) | 323 | 308 | 309 | 432 | 330 | 309 | 399 | 553 |
| PreBleed (08/20) | 212 | 209 | 151 | | 293 | 255 | 289 | |
| PreBleed (9/20) | 270 | 259 | 256 | 245 | 212 | 232 | 234 | 325 |
| PreBleed (9/20) | 323 | 282 | 212 | | 214 | 168 | | |
| PreBleed Wk-3 | 288 | 236 | 151 | | 217 | 222 | 153 | |
| PreBleed WK-1 | 389 | 378 | 333 | | 290 | 232 | 154 | |
| Wk 4 | 312 | 357 | 363 | | 340 | 316 | 328 | |
| Wk 6 | 1475 | 1682 | | | 1411 | 1734 | | |
| Wk 12 | 575 | 705 | 692 | | 553 | 671 | 743 | |
| Wk 14 | 2176 | | | | 1157 | 1292 | | |
| Wk 18 | 584 | 747 | 747 | | 550 | 728 | 779 | |
| Wk 20 | 581 | 748 | 920 | | 618 | 776 | 840 | |
| WK 22 | 1256 | | | | 462 | 976 | | |
| Wk 24 | 1104 | 1098 | | | 810 | 1072 | 1205 | |
| Wk 26 | 704 | 853 | 905 | | 688 | 729 | 923 | |

| NHP-4 | Series 1 | | | | Series 2 | | | |
|---|---|---|---|---|---|---|---|---|
| PreBleed (08/20) | 292 | 297 | 346 | 264 | 229 | 238 | 235 | 257 |
| PreBleed (08/20) | 174 | 106 | | | 150 | 81 | | |
| PreBleed (9/20) | 229 | 231 | 258 | 260 | 241 | 230 | 256 | 309 |
| PreBleed (9/20) | 258 | 154 | 186 | | 232 | 193 | 151 | |
| PreBleed (12/20) | 170 | | | | 189 | 89 | | |
| PreBleed (12/20) | 215 | | 186 | | 252 | 233 | 176 | 204 |
| Wk 4 | | | | | | | | |
| Wk 6 | 717 | 762 | | | 643 | 688 | 802 | |
| Wk 12 | 613 | 780 | 772 | | 585 | 658 | 796 | |
| Wk 14 | 1880 | 2040 | | | 1868 | 2200 | | |
| Wk 18 | 1007 | 1044 | | | 796 | 1089 | 1186 | |
| Wk 20 | 1160 | 1580 | | | 752 | 1104 | 880 | |
| Wk 22 | 1876 | | | | 605 | 1268 | | |
| Wk 24 | 709 | 906 | 907 | | 711 | 928 | 1011 | |
| Wk 26 | 2007 | 2295 | | | 2071 | 2235 | | |

| NHP-5 | Series 1 | | | | Series 2 | | | |
|---|---|---|---|---|---|---|---|---|
| PreBleed (08/20) | 130 | 44 | | | 136 | 59 | | |
| PreBleed (08/20) | 263 | 112 | | | 45 | | | |
| PreBleed (9/20) | 153 | 147 | 144 | 159 | 137 | 120 | 145 | 188 |
| PreBleed (9/20) | 194 | | | | 120 | | | |
| PreBleed (12/20) | 147 | 58 | | | 106 | 70 | | |
| PreBleed WK-1 | 120 | | | | 102 | | | |
| Wk 4 | 1185 | 1161 | | | 1161 | 1304 | | |
| Wk 6 | 1870 | 2276 | | | 1736 | 2405 | | |
| Wk 12 | 612 | 790 | 793 | | 571 | 745 | 743 | |
| Wk 14 | 1868 | 2040 | | | 1816 | 2220 | | |
| Wk 18 | 1199 | 1296 | | | 797 | 1300 | 1271 | |
| WK 20 | 758 | 1068 | 1060 | | 718 | 1052 | 1140 | |
| Wk 22 | 2064 | | | | 737 | 1204 | | |
| Wk 24 | 2139 | | | | 2212 | | | |
| Wk 26 | 1347 | 1500 | | | 846 | 1197 | 1377 | |

| NHP-6 | Series 1 | | | | Series 2 | | | |
|---|---|---|---|---|---|---|---|---|
| PreBleed (08/20) | 147 | 88 | | | 120 | 64 | | |
| PreBleed (08/20) | | | | 372 | 159 | 419 | 200 | |
| PreBleed (09/20) | 156 | 151 | 137 | 138 | 143 | 144 | 130 | 188 |
| PreBleed (12/20) | 53 | 139 | 254 | | 279 | | | |
| PreBleed WK-1 | 166 | | | | 411 | | 187 | |
| PreBleed Wk0 | 62 | 217 | 271 | | | | | |
| Wk 4 | 755 | 824 | 882 | | 655 | 763 | 889 | |
| Wk 6 | 929 | 432 | | | 1042 | 1296 | | |
| Wk 12 | 661 | 794 | 739 | | 635 | 780 | 776 | |
| Wk 14 | 1672 | 1740 | | | 1492 | 1880 | | |
| Wk 18 | 708 | 799 | 732 | | 651 | 735 | 876 | |
| WK 20 | 1064 | 1080 | | | 745 | 1100 | 1260 | |
| WK 22 | 912 | | | | 399 | 576 | | |
| Wk 24 | 1732 | 1942 | | | 1584 | 2056 | | |
| Wk 26 | 1189 | 1343 | | | 1230 | 1564 | | |

| NHP-7 | Series 1 | | | | Series 2 | | | |
|---|---|---|---|---|---|---|---|---|
| PreBleed (08/20) | 195 | 156 | 161 | 148 | 186 | 233 | 169 | 278 |
| PreBleed (08/20) | 90 | | | | 111 | 2 | | |
| PreBleed (09/20) | 292 | 269 | 255 | 262 | 274 | 375 | 223 | 309 |
| PreBleed (12/20) | 219 | 193 | | | 210 | | | |
| PreBleed (12/20) | 202 | 159 | | | 214 | 446 | | |
| PreBleed Wk0 | 107 | | | | 150 | 283 | 487 | |
| Wk 4 | 437 | 508 | 529 | | 424 | 494 | 598 | |
| Wk 6 | 1526 | 1611 | | | 1548 | 1377 | | |
| Wk 12 | 579 | 521 | | | 554 | 534 | 612 | |
| WK 14 | 2272 | 2840 | | | 2364 | 3040 | | |
| Wk 18 | 637 | 678 | 639 | | 577 | 740 | 794 | |
| WK 20 | 1440 | | | | 602 | 1324 | | |
| Wk 22 | 1348 | | | | 530 | 548 | | |
| Wk 24 | 772 | 946 | 1012 | | 570 | 916 | 1164 | |
| Wk 26 | 1185 | 1339 | | | 1293 | 1366 | | |
